# Supplementary material for: LRP5 promotes adipose progenitor cell fitness and adipocyte insulin sensitivity
Source: Commun Med (Lond). 2025 Feb 25;5:51. doi: 10.1038/s43856-025-00774-1 (PMC11862225; doi:10.1038/s43856-025-00774-1)
Supplement: Supplementary file 2 — Supplementary Information [file 43856_2025_774_MOESM2_ESM.pdf]

Supplementary Information

LRP5 promotes adipose progenitor cell fitness and adipocyte insulin sensitivity.

Nellie Y. Loh, Senthil K. Vasan, Daniel B. Rosoff , Emile Roberts, Andrea D. van Dam, Manu Verma, Daniel Phillips, Agata Wesolowska-Andersen, Matt J. Neville, Raymond Noordam, David W. Ray, Jonathan H. Tobias, Celia L. Gregson, Fredrik Karpe and Constantinos Christodoulides.

SUPPLEMENTARY FIGURES

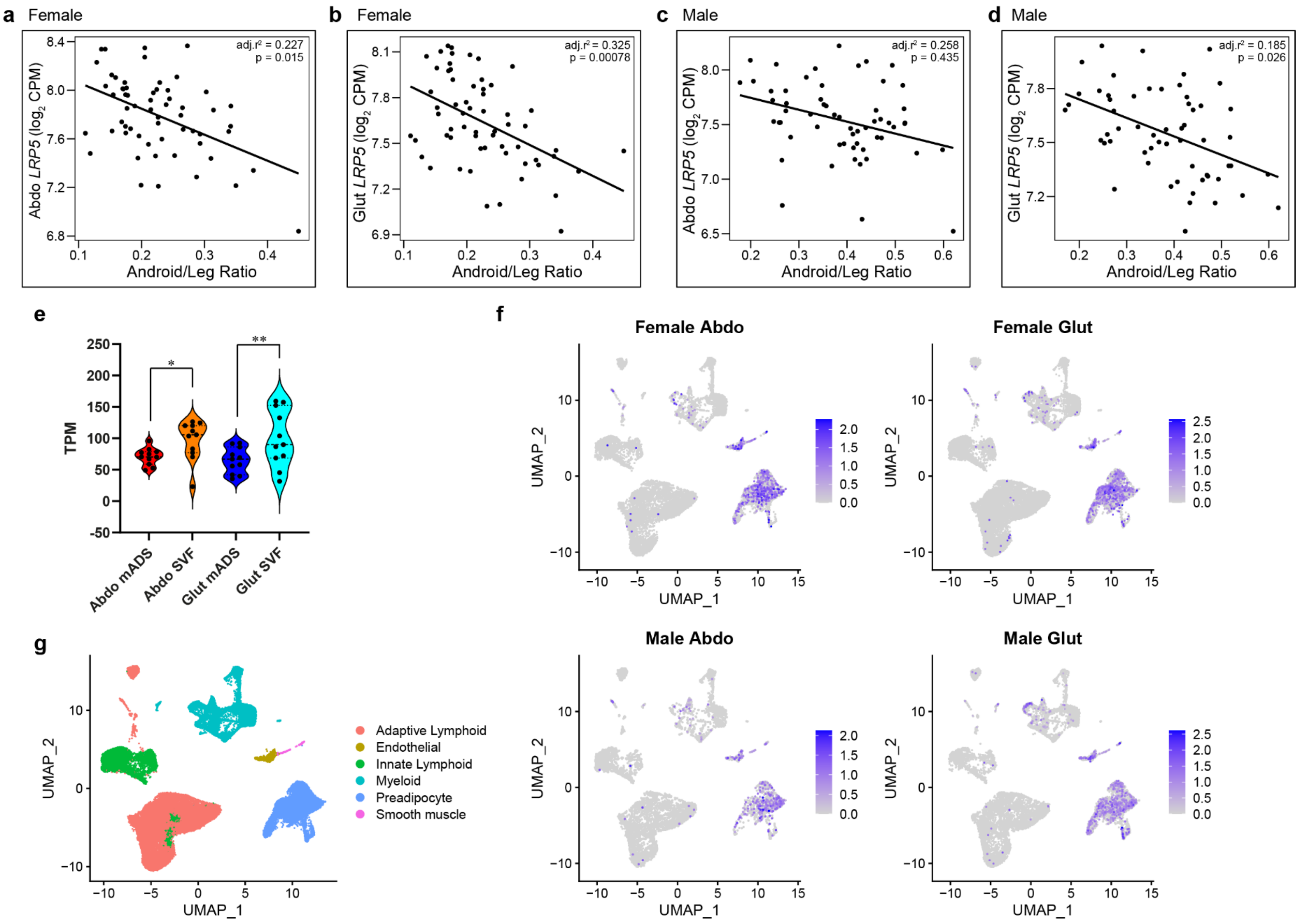

Supplementary Fig. 1. *LRP5* expression in human subcutaneous white adipose tissue (WAT) and WAT fractions. (**a**-**d**) *LRP5* expression in abdominal and gluteal subcutaneous WAT correlates negatively with android/leg fat ratio. Linear regression of *LRP5* expression in abdominal (Abdo) (**a**, **c**) and gluteal (Glut) (**b**, **d**) subcutaneous WAT and android/leg fat ratios in (**a**, **b**) women (n = 61, age = 50 ± 6.2 years, BMI = 25.9 ± 3.2 kg/m^2) and (**c**, **d**) men (n = 59, age = 48 ± 7.7 years, BMI = 26.7 ± 3.3 kg/m^2). r^2 and p-values shown are for results adjusted for age, fat mass index (fatmass [kg]/(height [m])^2), and batch. (**e**) *LRP5* expression in fractionated WAT. mADS, mature adipocytes; SVF, stromovascular fraction. RNA-sequencing data from 11 females. TPM, transcripts per million. *p <0.05, **p ≤0.01, two-tailed paired Student's t-test. (**f**) Single cell RNA-sequencing feature plots for *LRP5* expression in subpopulations of SVF of subcutaneous abdominal and gluteal WAT from 15 men (age = 47.7 ± 6.2 years, BMI = 25.9 ± 1.4 kg/m^2) and 17 women (age = 45.9 ± 3.8 years, BMI = 25.2 ± 1.5 kg/m^2). (**g**) Unsupervised clustering of stromovascular cell populations shown as uniform manifold approximation and projection (UMAP).

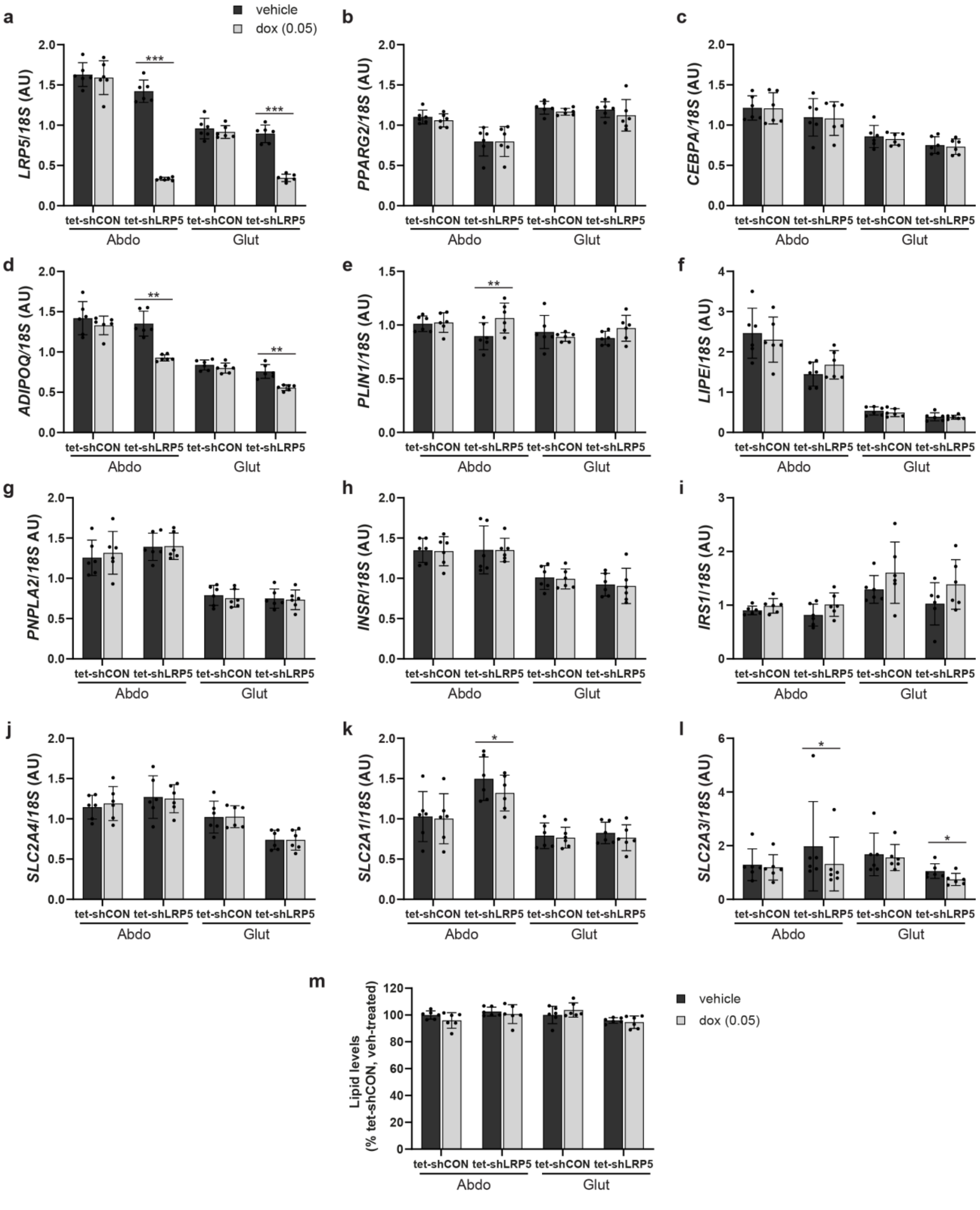

Supplementary Fig. 2. mRNA expression analyses of adipogenic and insulin signaling pathway genes and AdipoRed assay of *in vitro* differentiated DFAT tet-shCON and tet-

shLRP5 stable cell lines. (**a-l**) DFAT cells were harvested for RNA at day 15 of adipogenic differentiation following ~48 hours treatment with 0.05µg/ml doxycycline or vehicle in hormone-free basal media (n = 6 experiments). qRT-PCR data were normalised to *18S.* (**a**) (genotype x dox)$_{Abdo}$ p <0.0001; (genotype x dox)$_{Glut}$ p = 0.0002; (**d**) (genotype x dox)$_{Abdo}$ p = 0.02; (genotype x dox)$_{Glut}$ p = 0.03; (**e**) (genotype x dox)$_{Abdo}$ p = 0.01; (**k**) (genotype x dox)$_{Abdo}$ p = 0.048; (**l**) (genotype x dox)$_{Abdo}$ p = 0.052; (genotype x dox)$_{Glut}$ p = 0.21. (**m**) Quantification of AdipoRed staining of *in vitro* differentiated d15 cells following ~48-hour treatment with vehicle or 0.05 µg/ml doxycycline (n = 6 replicates). Histograms are means ± SD. *p <0.05, **p <0.01, ***p <0.0001. Statistical significance was determined by (**a-l**) 2-way repeated measures ANOVA, and (**m**) 2-way ANOVA, with Sidak's multiple comparisons test comparing doxycycline and vehicle-treated groups.

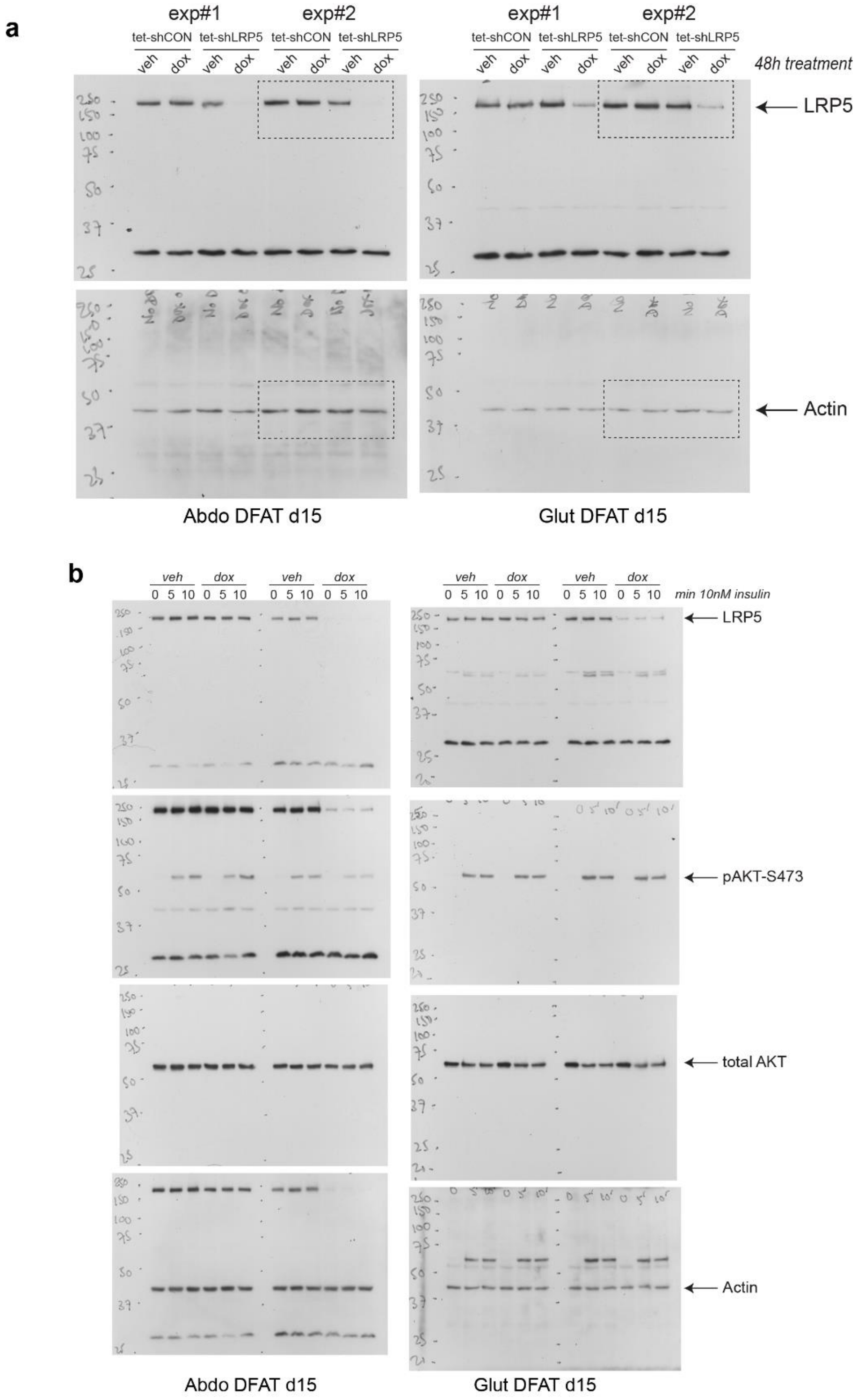

Supplementary Fig. 3. Unprocessed scans for Fig. 2. (**a**) Unedited Western blots for Fig. 2c. The replicates used in the figure are boxed. (**b**) Unedited Western blots for Fig. 2h. Actin was used as loading control.

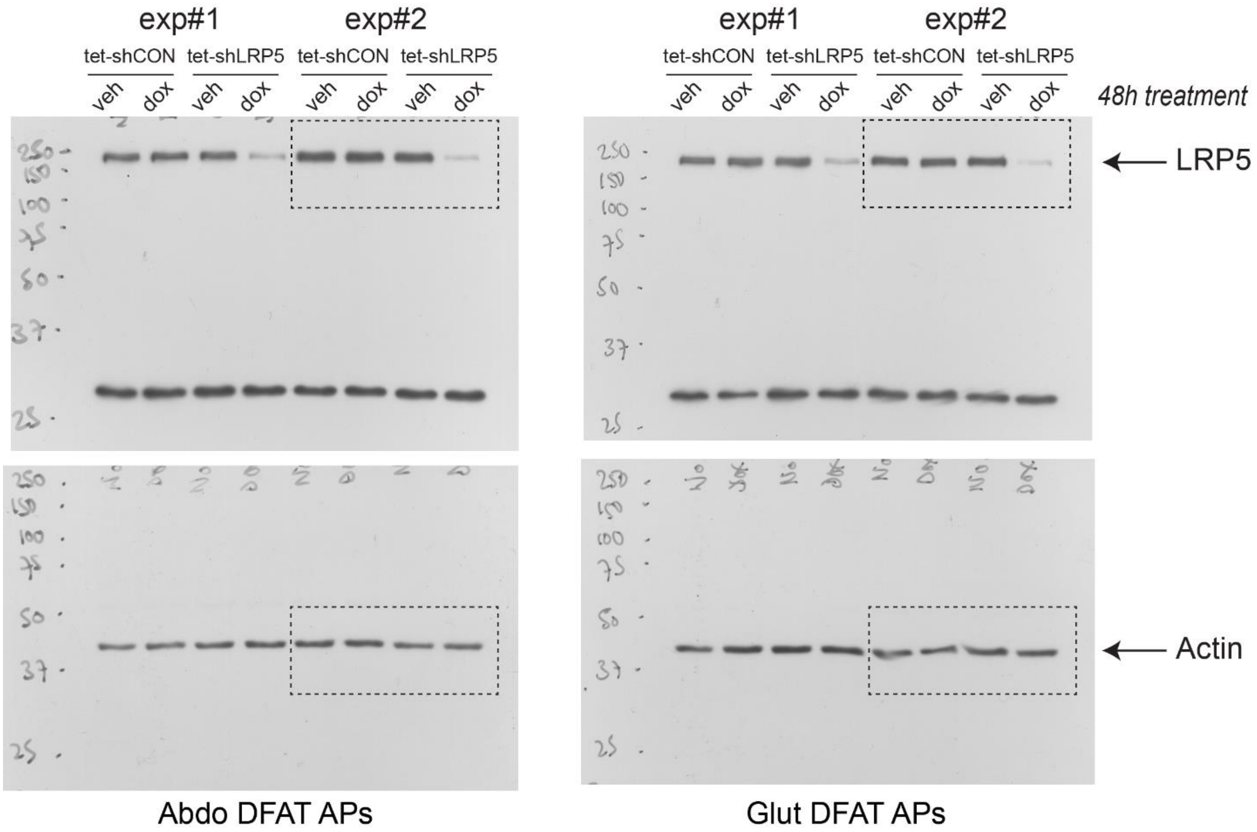

Supplementary Fig. 4. Unprocessed scans for Fig. 3. Unedited Western blots for Fig. 3b. The replicates used in the figure are boxed. Actin was used as loading control.

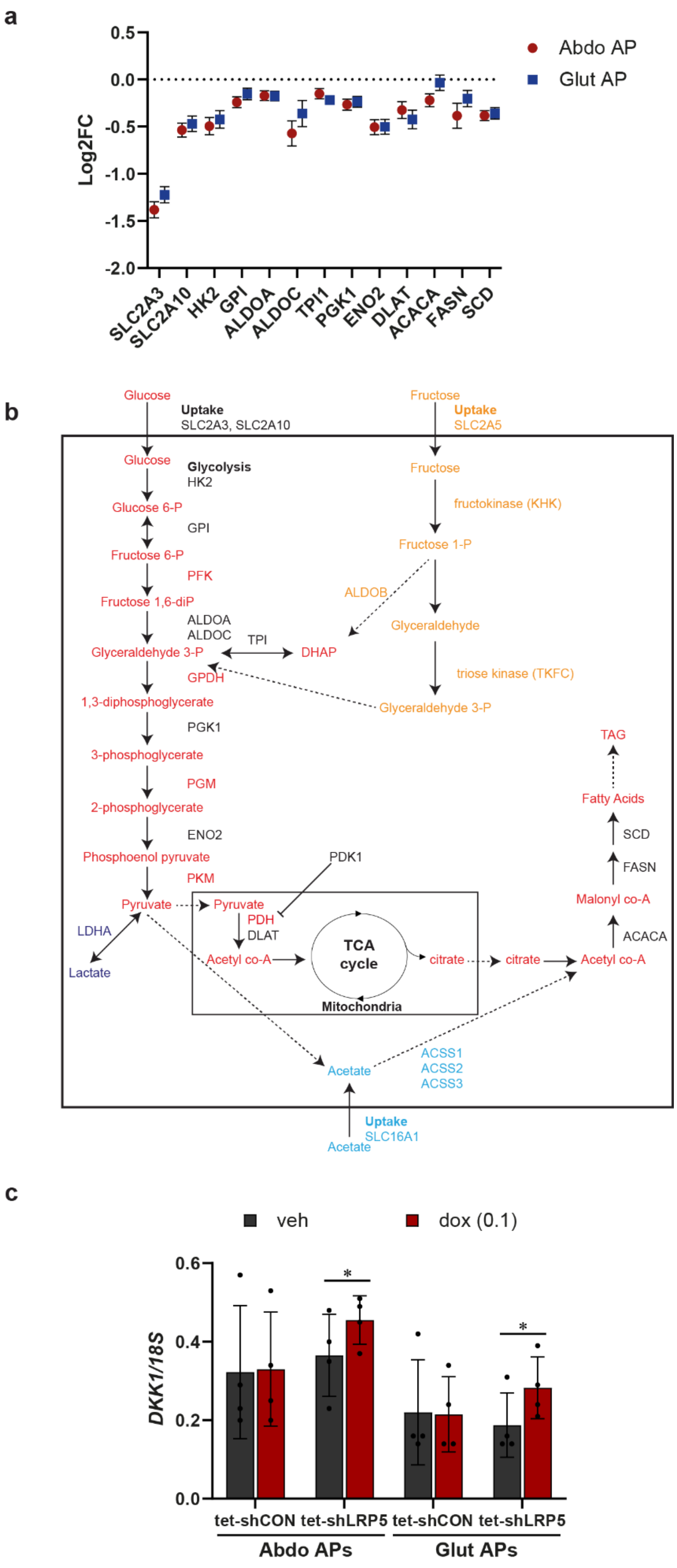
a
Abdo AP
Glut AP
Log2FC
0.5
0.0
-0.5
-1.0
-1.5
-2.0
SLC2A3
SLC2A10
HK2
GPI
ALDOA
ALDOC
TPI1
PGK1
ENO2
DLAT
ACACA
FASN
SCD
b
Glucose
Uptake
SLC2A3, SLC2A10
Glucose
Glycolysis
HK2
Glucose 6-P
GPI
Fructose 6-P
PFK
Fructose 1,6-diP
ALDOA
ALDOC
TPI
Glyceraldehyde 3-P
DHAP
GPDH
1,3-diphosphoglycerate
PGK1
3-phosphoglycerate
PGM
2-phosphoglycerate
ENO2
Phosphoenol pyruvate
PKM
Pyruvate
LDHA
Lactate
Fructose
Uptake
SLC2A5
Fructose
fructokinase (KHK)
Fructose 1-P
ALDOB
Glyceraldehyde
triose kinase (TKFC)
Glyceraldehyde 3-P
TAG
Fatty Acids
SCD
FASN
Malonyl co-A
ACACA
PDK1
Pyruvate
PDH
DLAT
Acetyl co-A
TCA cycle
citrate
citrate
Acetyl co-A
Mitochondria
Acetate
ACSS1
ACSS2
ACSS3
Uptake
SLC16A1
Acetate
c
veh
dox (0.1)
DKK1/18S
0.6
0.4
0.2
0.0
*
*
tet-shCON
tet-shLRP5
tet-shCON
tet-shLRP5
Abdo APs
Glut APs

Supplementary Fig. 5. Doxycycline-induced *LRP5*-KD in APs, glycolysis, and DKK1. (**a**) *LRP5*-KD downregulates multiple genes in the glycolysis pathway. RNA-seq data from 3 independent experiments, padj <0.05. (**b**) Schematic of the glycolysis pathway. Differentially expressed genes in black font. ACACA, acetyl-coA calboxylase alpha; ACSS, acyl-coA synthetase short chain; ALDO, aldolase; DLAT, dihydrolipoamide S-acetyltransferase; ENO, enolase; FASN, fatty acid synthase; GPDH, glyceraldehyde 3-phospate dehydrogenase; GPI, phosphohexose isomerase; HK2, hexokinase 2; LDHA, lactate dehydrogenase A; PDH, pyruvate dehydrogenase; PDK1, pyruvate dehydrogenase kinase 1; PFK, phosphofructokinase; PGK, phosphoglycerate kinase; PGM, phosphoglycerate mutase; PKM, pyruvate kinase M1/2; SCD, stearoyl-coA desaturase; SLC2A3, glucose transporter GLUT3; SLC2A10, glucose transporter GLUT10; SLC2A5, fructose transporter; SLC16A1, monocarboxylate transporter; TPI, triosephosphate isomerase. (**c**) *LRP5*-KD upregulates *DKK1* expression in APs (n = 4 experiments, $(\text{genotype x dox})_{\text{Abdo}}$ $p = 0.03$; $(\text{genotype x dox})_{\text{Glut}}$ $p = 0.02$). qRT-PCR data were normalised to *18S.* Histogram are means ± SD. *$p < 0.05$, two-way repeated measures ANOVA with Sidak's multiple comparisons test.

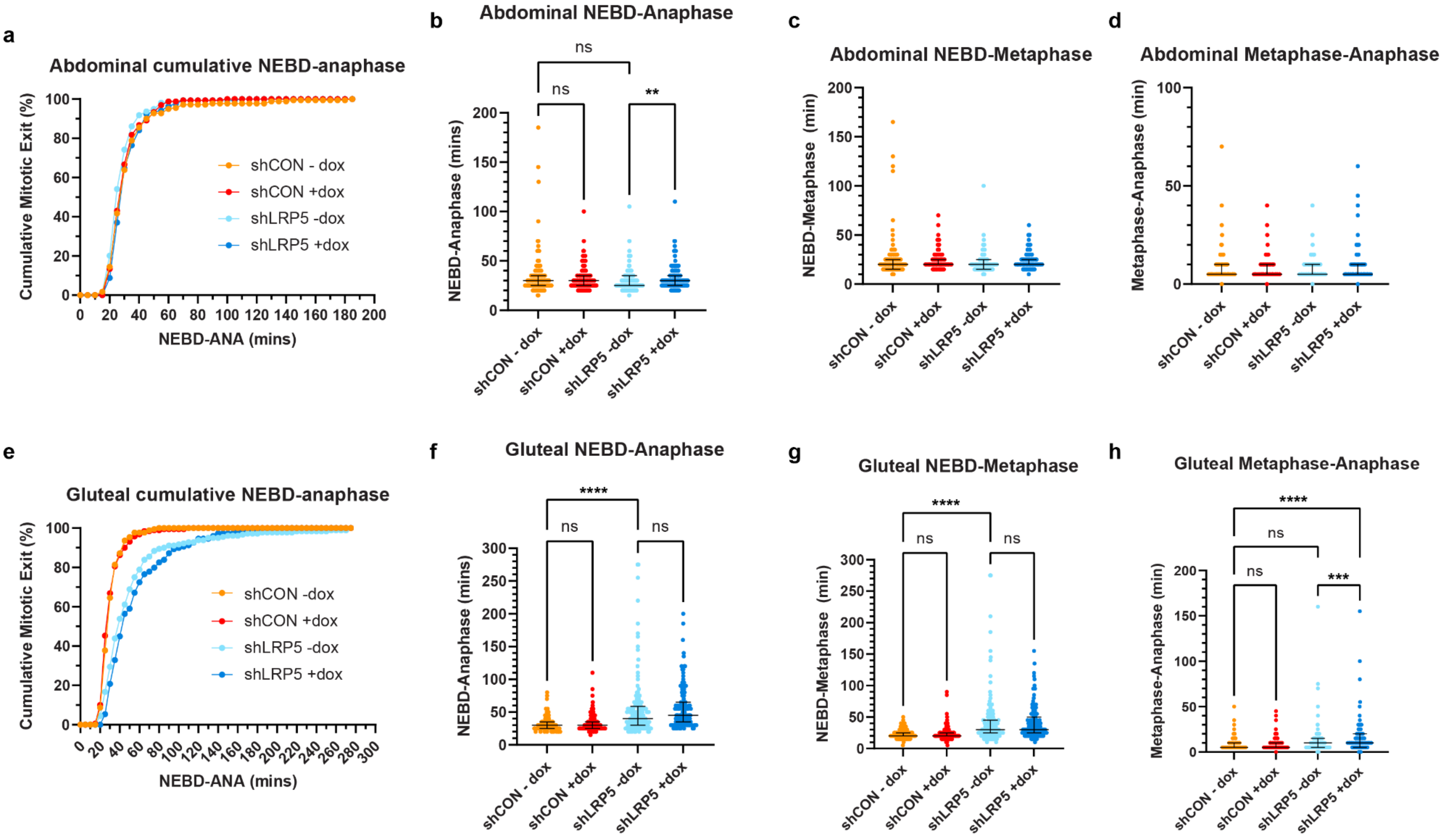

Supplementary Fig. 6. Mitotic timings of control and *LRP5*-KD abdominal (a-d) and gluteal (e-h) DFAT APs. Cells were synchronized at S-phase using a single 17h thymidine block. Seven hours after thymidine washout, and 1 hour after addition of SiR-

DNA stain, cells underwent live cell imaging (at 5-min intervals for 12 hours). Live cell imaging data were used to quantify the times at which nuclear envelope breakdown (NEBD), metaphase, and anaphase occurred based on DNA morphology. (**a, e**) Cumulative (%) cells at mitotic exit (NEBD to anaphase). Scatter plots showing the time taken for cells to transition from (**b, f**) NEBD to anaphase, (**c, g**) NEBD to metaphase, and (**d, h**) metaphase to anaphase. Statistics: Kruskal-Wallis with Dunn's multiple comparisons test. Results from 3 independent experiments. $^{**}p < 0.01$, $^{***}p < 0.001$, $^{****}p < 0.0001$.

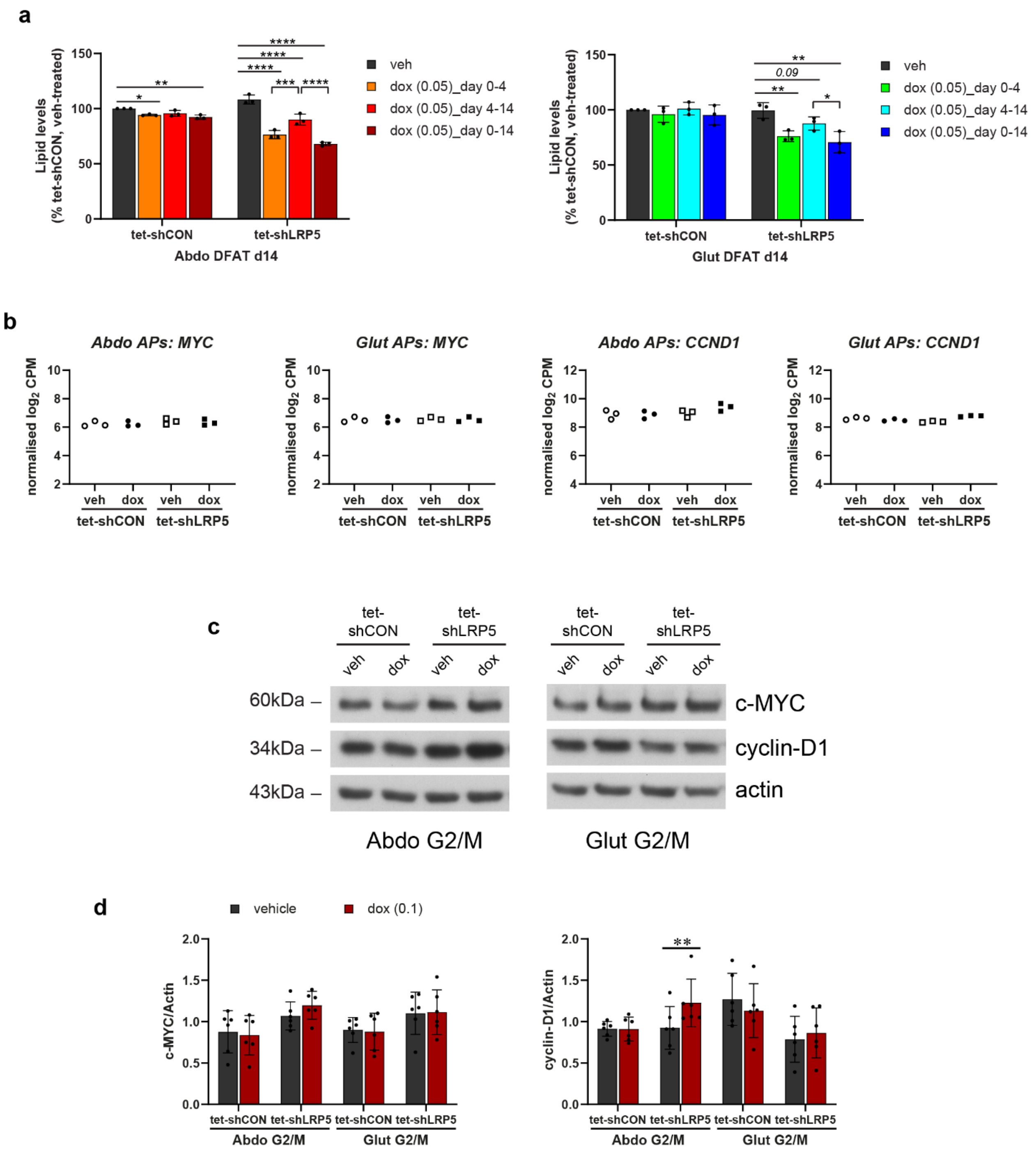

Supplementary Fig. 7. Effects of doxycycline-induced *LRP5*-KD on abdominal and gluteal AP biology. (**a**) Effect of *LRP5*-KD at different stages of adipogenesis on lipid accumulation in abdominal and gluteal cells (n = 3 independent experiments). **p* <0.05, ***p* <0.01, *****p* <0.0001,

two-way repeated measures ANOVA with Tukey's multiple comparisons test. (genotype x treatment)$_{Abdo}$ p <0.0001; (genotype x treatment)$_{Glut}$ p = 0.02. (**b**) Gene expression of *MYC* and *CCND1*. Data from RNA-seq. (**c**) Western blots of c-MYC and cyclin-D1 in vehicle and doxycycline-treated G2/M arrested cells. (**d**) Normalized protein densitometry for c-MYC and cyclin-D1 from G2/M arrested cells (n = 6) (cyclin-D1: (genotype x dox)$_{Abdo}$ p = 0.009). **p <0.01, two-way repeated measures ANOVA with Sidak's multiple comparisons test. Histograms are means ± SD. Actin was used as loading control for Western blots.

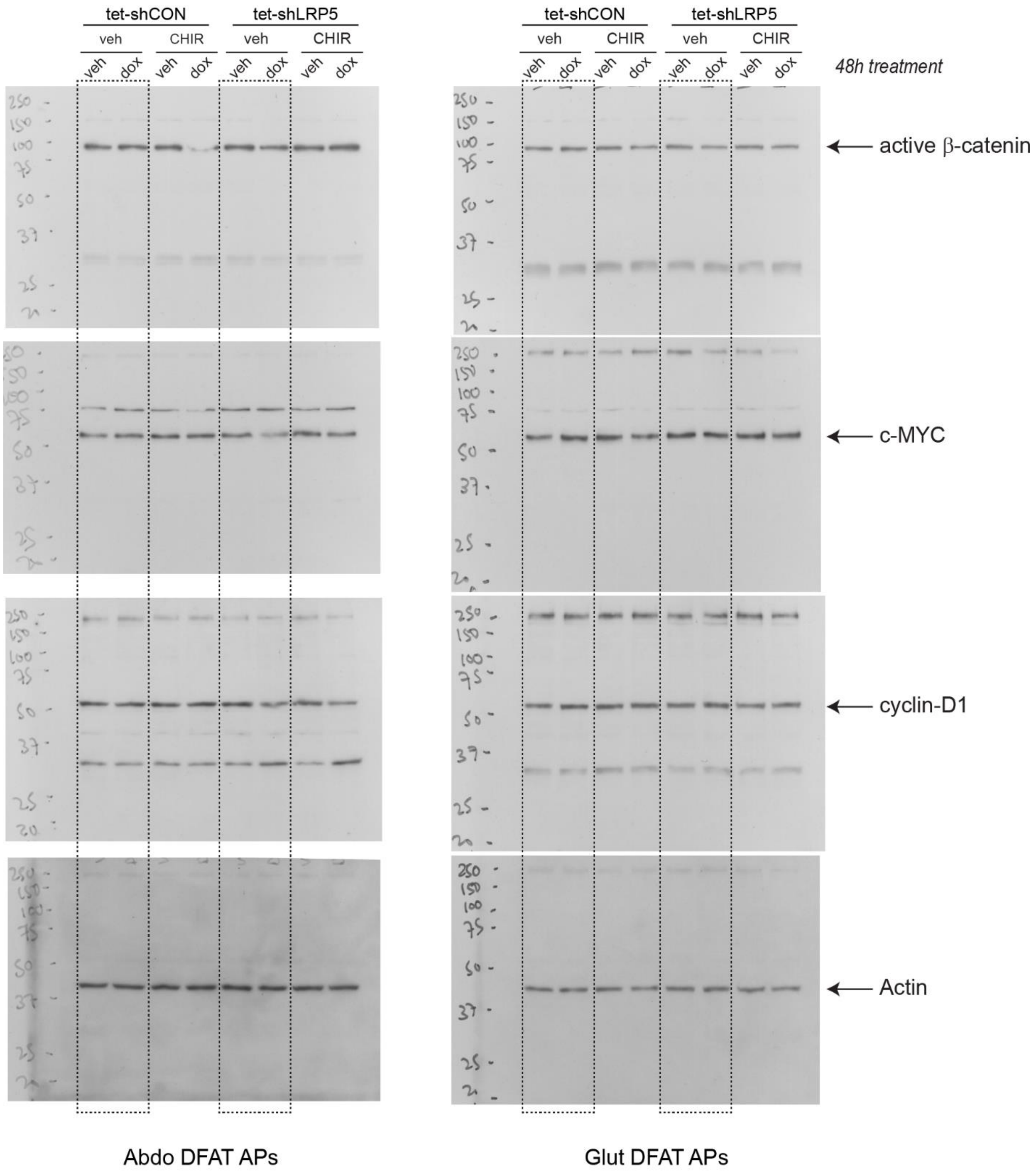

Supplementary Fig. 8. Unprocessed scans for Fig. 4. Unedited Western blots for Fig. 4e. The lanes of the blots used in the figure are boxed. Actin was used as loading control.

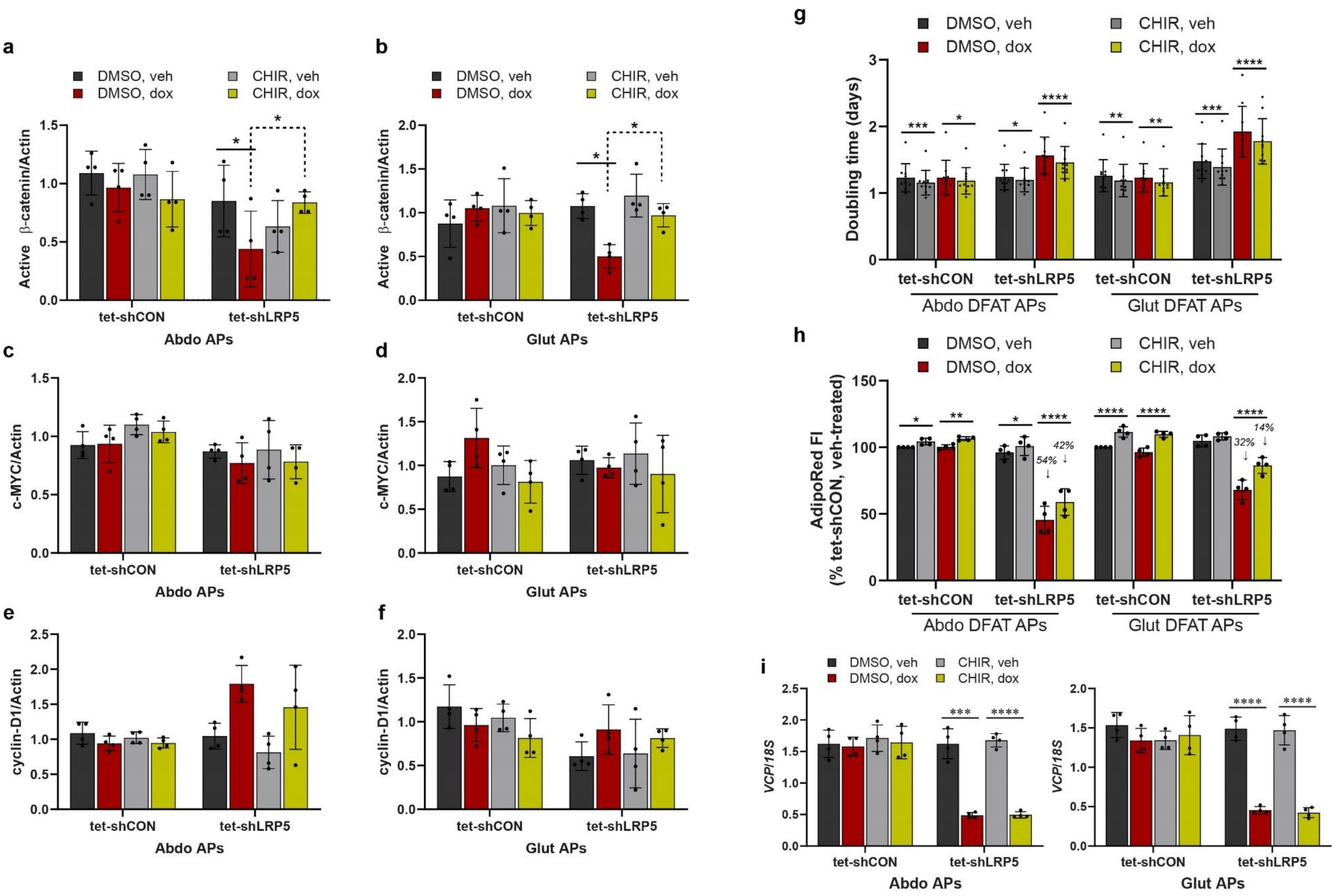

a
DMSO, veh
DMSO, dox
CHIR, veh
CHIR, dox
Active β-catenin/Actin
tet-shCON
tet-shLRP5
Abdo APs
b
Glut APs
c
c-MYC/Actin
d
e
cyclin-D1/Actin
f
g
Doubling time (days)
Abdo DFAT APs
Glut DFAT APs
h
AdipoRed FI (% tet-shCON, veh-treated)
54%
42%
32%
14%
i
VCP/18S

Supplementary Fig. 9. Treatment with a GSK3 inhibitor, CHIR99021, is not able to prevent defects in DFAT cells due to *LRP5*-KD. (**a**-**f**) Treatment with CHIR99021 normalizes active β-catenin expression, but does not affect c-MYC or cyclin-D1 expression in control and *LRP5*-KD Abdo and Glut APs. Protein densitometry of (**a**-**b**) active β-catenin [(dox x CHIR99021)$_{\text{Abdo tet-shLRP5}}$ $p = 0.005$; (dox x CHIR99021)$_{\text{Glut tet-shLRP5}}$ $p = 0.04$)], (**c**-**d**) c-MYC and (**e**-**f**) cyclin-D1 expression are normalized to actin. N = 4 experiments. (**g**-**h**) Treatment with CHIR99021 partially rescues inhibition in proliferation and adipogenesis due to *LRP5*-KD in Abdo and Glut DFAT APs. Cells treated with vehicle (veh) or doxycycline in the presence of DMSO or 0.5µM CHIR99021 were assessed for (**g**) doubling time (n = 10; (genotype/dox x CHIR99021)$_{\text{Abdo}}$ $p = 0.04$; (genotype/dox x CHIR99021)$_{\text{Glut}}$ $p = 0.02$), (**h**) adipogenesis (n = 4 four independent experiments; (genotype/dox x CHIR99021)$_{\text{Abdo}}$ $p = 0.002$; (genotype/dox x CHIR99021)$_{\text{Glut}}$ $p < 0.0001$). CHIR99021 was included throughout the 14-day adipogenesis protocol. % in reduction in lipid staining relative to DMSO/vehicle-treated tet-shCON cells are shown. (**i**) Treatment with CHIR99021 does not rescue *VCP* expression in *LRP5*-KD Abdo and Glut APs. qRT-PCR of *VCP* in tet-shCON and tet-shLRP5 DFAT APs following ~2-day treatment with vehicle or 0.1µg/ml doxycycline in the presence of DMSO or 0.5µM CHIR99021 (n = 4 independent experiments; (genotype x dox x CHIR99021)$_{\text{Abdo}}$ $p = 0.92$; (genotype x dox x CHIR99021)$_{\text{Glut}}$ $p = 0.15$). Statistical analyses: (**a**-**f**) two-way RM ANOVA, with Tukey's multiple comparisons test, (**g-h**) two-way RM ANOVA with Sidak's multiple comparisons test, (**i**) three-way RM ANOVA with Sidak's multiple comparisons test. **p* <0.05, ***p* <0.01, ****p* <0.001, *****p* <0.0001. Histograms are means ± SD.

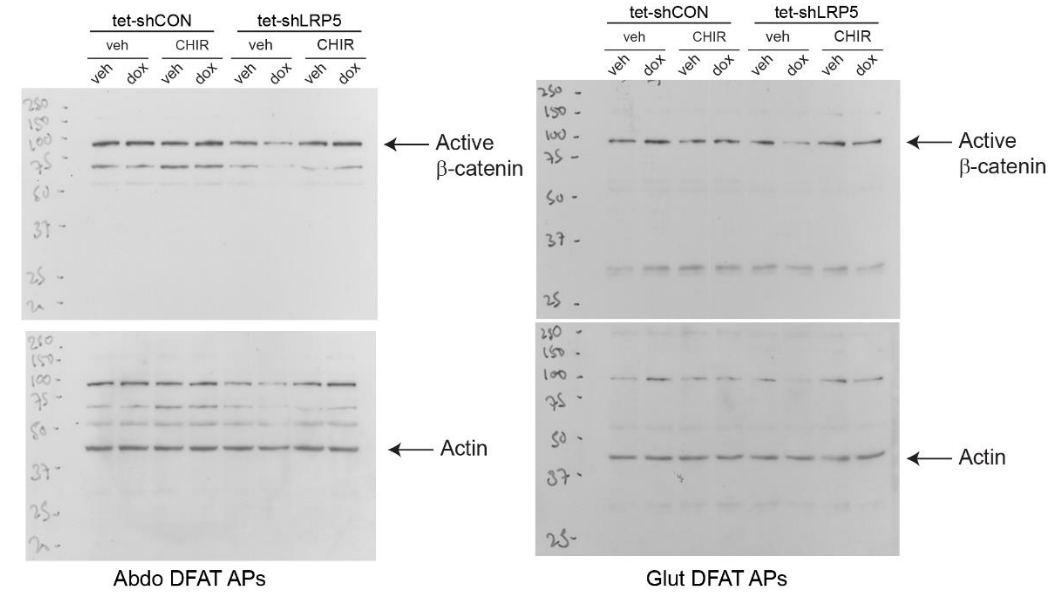

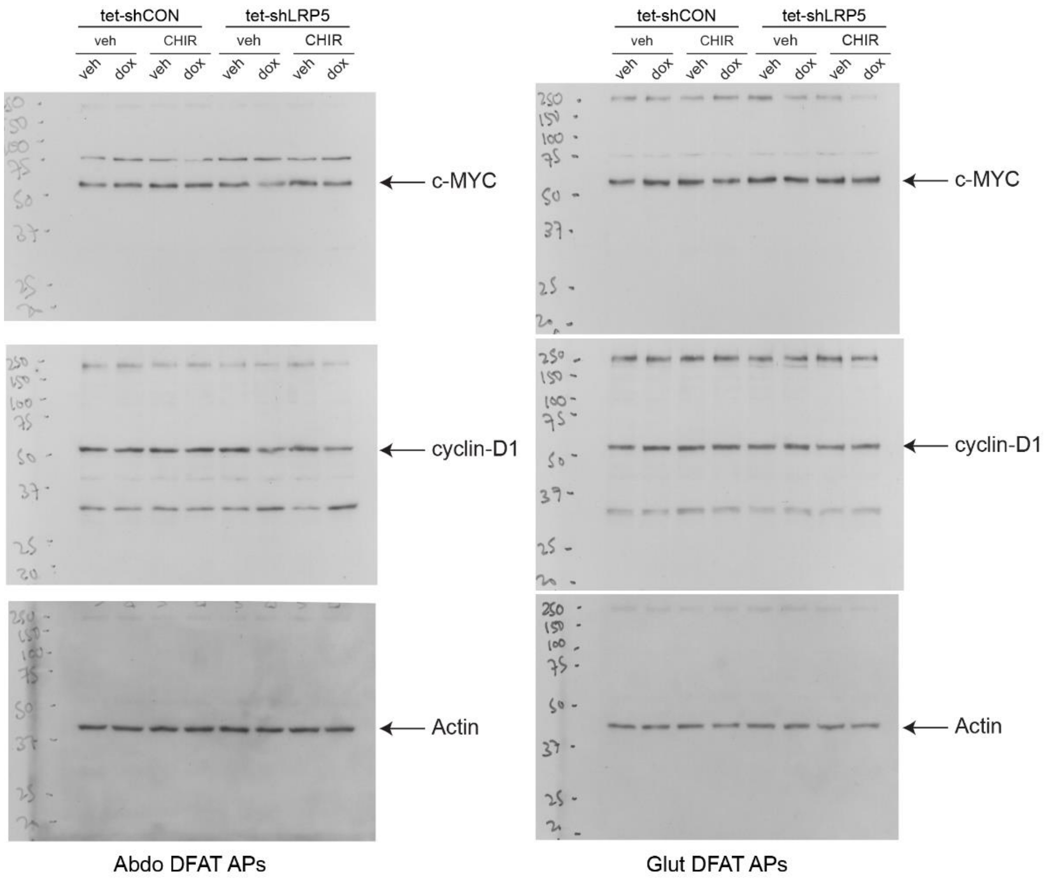

Supplementary Fig. 10. Unprocessed scans for Fig. 5. Unedited Western blots for Fig. 5k. Actin was used as loading control.

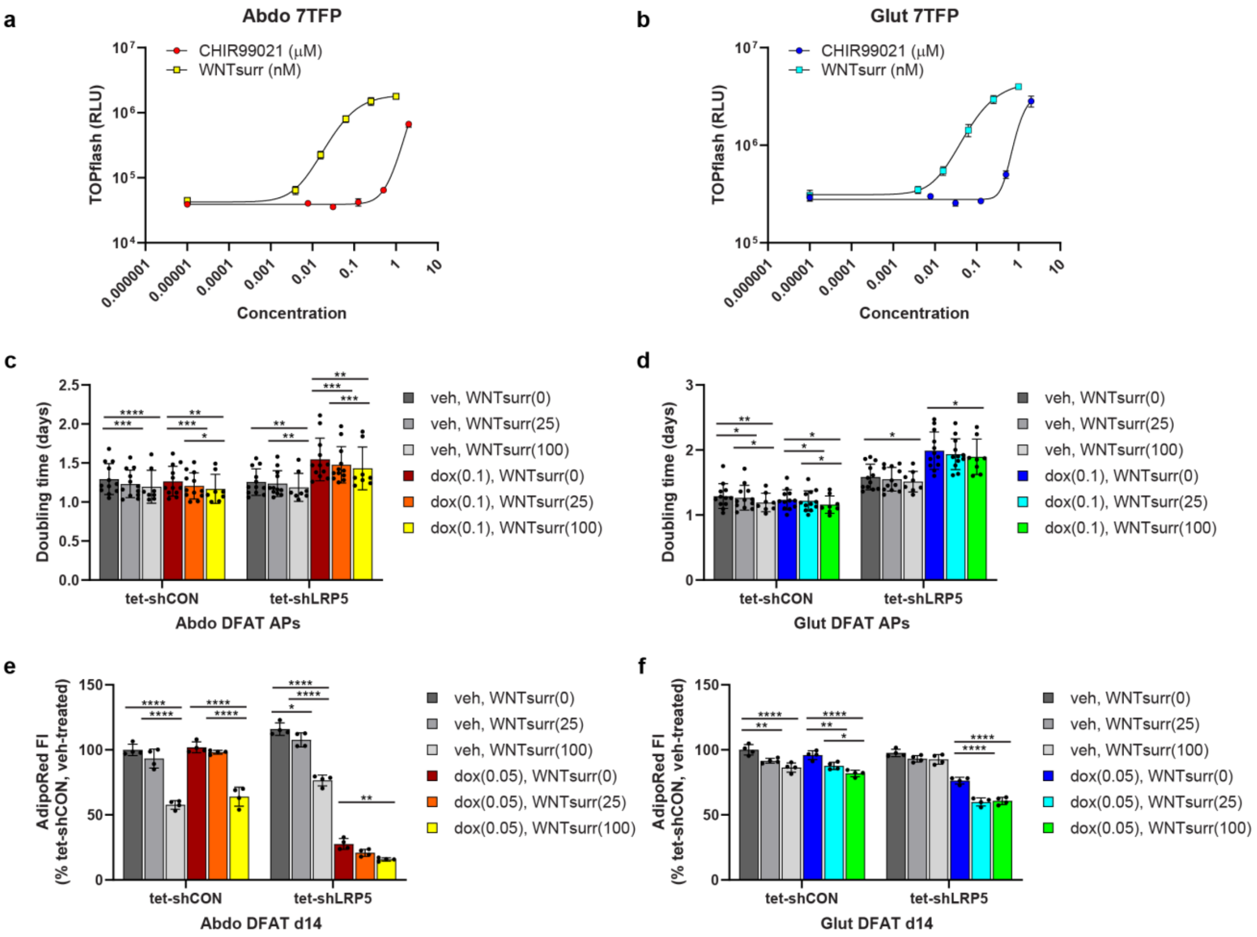

Supplementary Fig. 11. Treatment with a WNT surrogate-Fc fusion recombinant protein (WNTsurr) is able to partially rescue inhibition of proliferation, but not adipogenesis, in DFAT *LRP5*-KD cells. (**a-b**) Dose response curve of WNTsurr and CHIR99021 on TOPflash activity in immortalized abdominal (**a**) and gluteal (**b**) APs (data shown as mean ± SD, n = 4). (**c-f**) Effects of WNTsurr treatment (25pM and 100pM) on (**c-d**) doubling time (n = 12 experiments for vehicle- and 25pM WNTsurr-treated cells, n = 8 experiments for 100pM WNTsurr-treated cells; Fixed effects type III, (genotype/dox x WNTsurr)$_{Abdo}$ p = 0.16; (genotype/dox x WNTsurr)$_{Glut}$ p = 0.35), and (**e**-**f**) adipogenesis (n = 4, representative of two independent experiments; (genotype/dox x WNTsurr)$_{Abdo}$ p <0.0001; (genotype/dox x WNTsurr)$_{Glut}$ p =0.002). *p <0.05, **p <0.01, ***p <0.001, ****p <0.0001. Statistics: (**c**, **d**) Mixed-effects analysis with Tukey's multiple

comparisons test; (**e**, **f**) two-way ANOVA with Tukey's multiple comparison's test. Histograms are means ± SD.

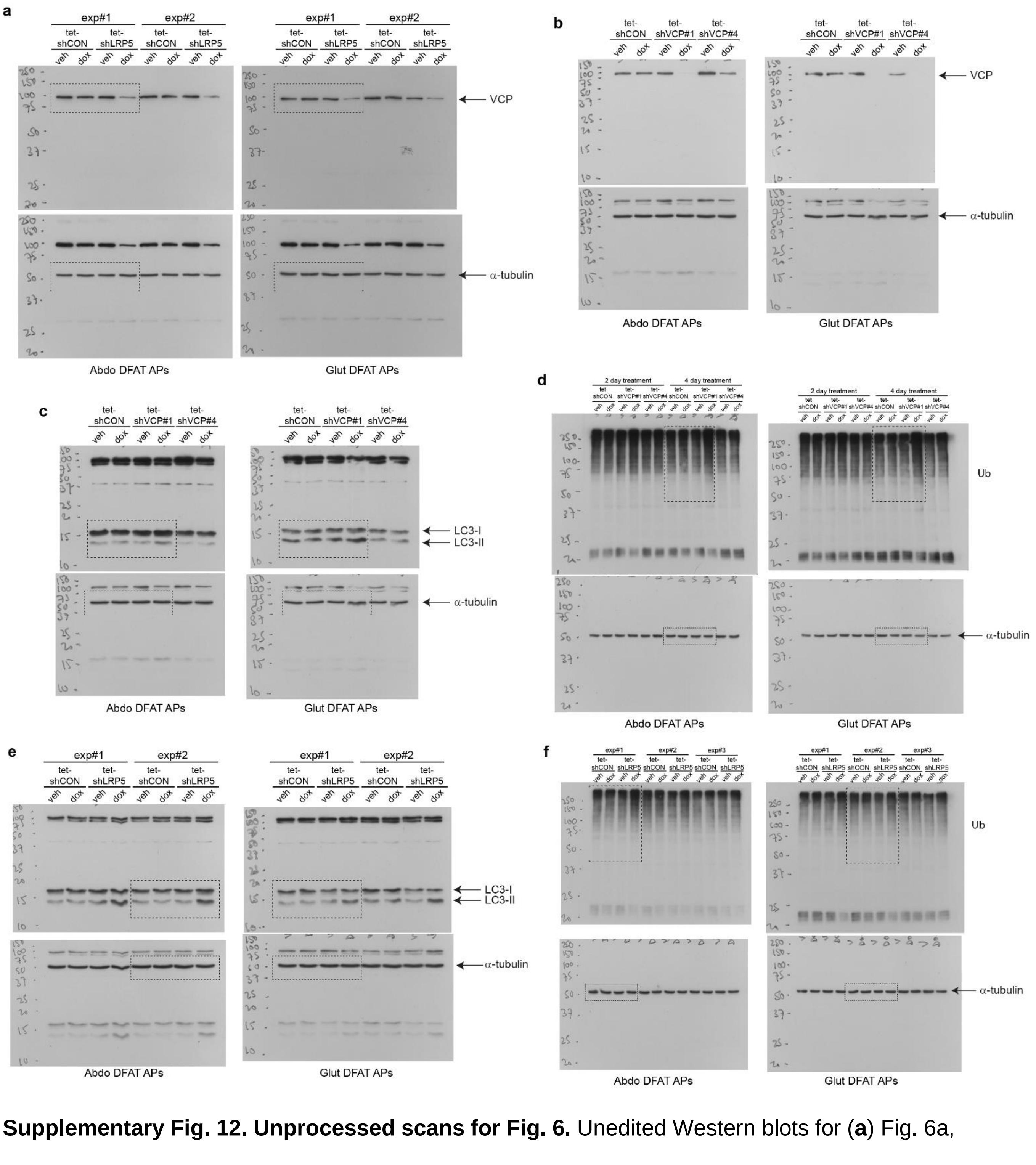

Supplementary Fig. 12. Unprocessed scans for Fig. 6. Unedited Western blots for (**a**) Fig. 6a, (**b**) Fig. 6d, (**c**) Fig.6g, (**d**) Fig. 6h, (**e**) Fig. 6i and (**f**) Fig. 6j. The replicates used in the figure are boxed. α-tubulin was used as loading control.

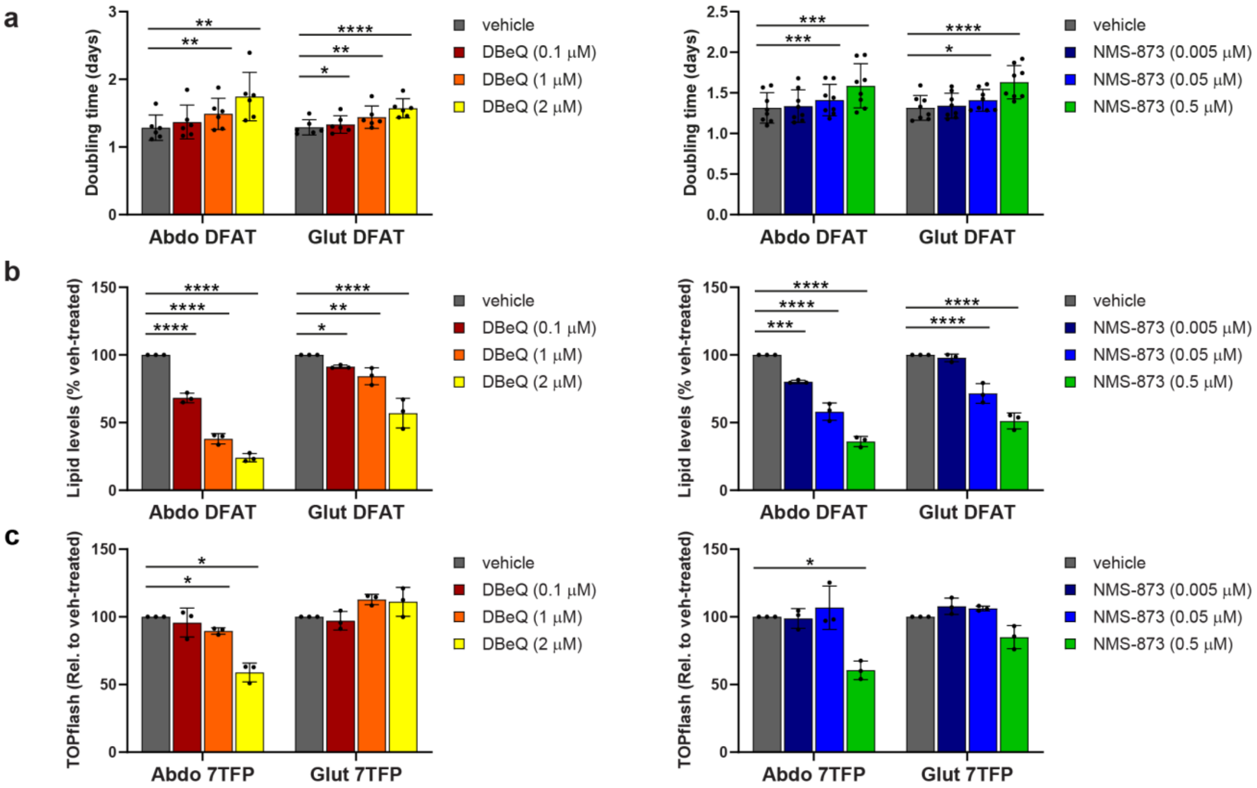

Supplementary Fig. 13. Effects of VCP inhibitor treatment on Abdo and Glut DFAT AP biology and TOPflash reporter activity. Effects of VCP inhibitors on cell: (**a**) doubling time (n = 6 (DBeQ) and 8 (NMS-873) independent experiments, (cell line x treatment)$_{DBeQ}$ $p = 0.03$; (cell line x treatment)$_{NMS\text{-}873}$ $p = 0.19$), and (**b**) adipogenesis (n = 3 independent experiments, (cell line x treatment)$_{DBeQ}$ $p < 0.0001$; (cell line x treatment)$_{NMS\text{-}873}$ $p = 0.005$). (**c**) Effects of VCP inhibitors, DBeQ and NMS-873, on TOPflash activity in immortalised APs (n = 3 independent experiments, (cell line x treatment)$_{DBeQ}$ $p = 0.01$; (cell line x treatment)$_{NMS\text{-}873}$ $p = 0.15$). *$p < 0.05$, **$p < 0.01$, ***$p < 0.001$, ****$p < 0.0001$, vehicle *vs*. DBeQ or NMS873-treatment. Statistical analyses: two-way RM ANOVA with Tukey's multiple comparisons test. Histograms are means ± SD.

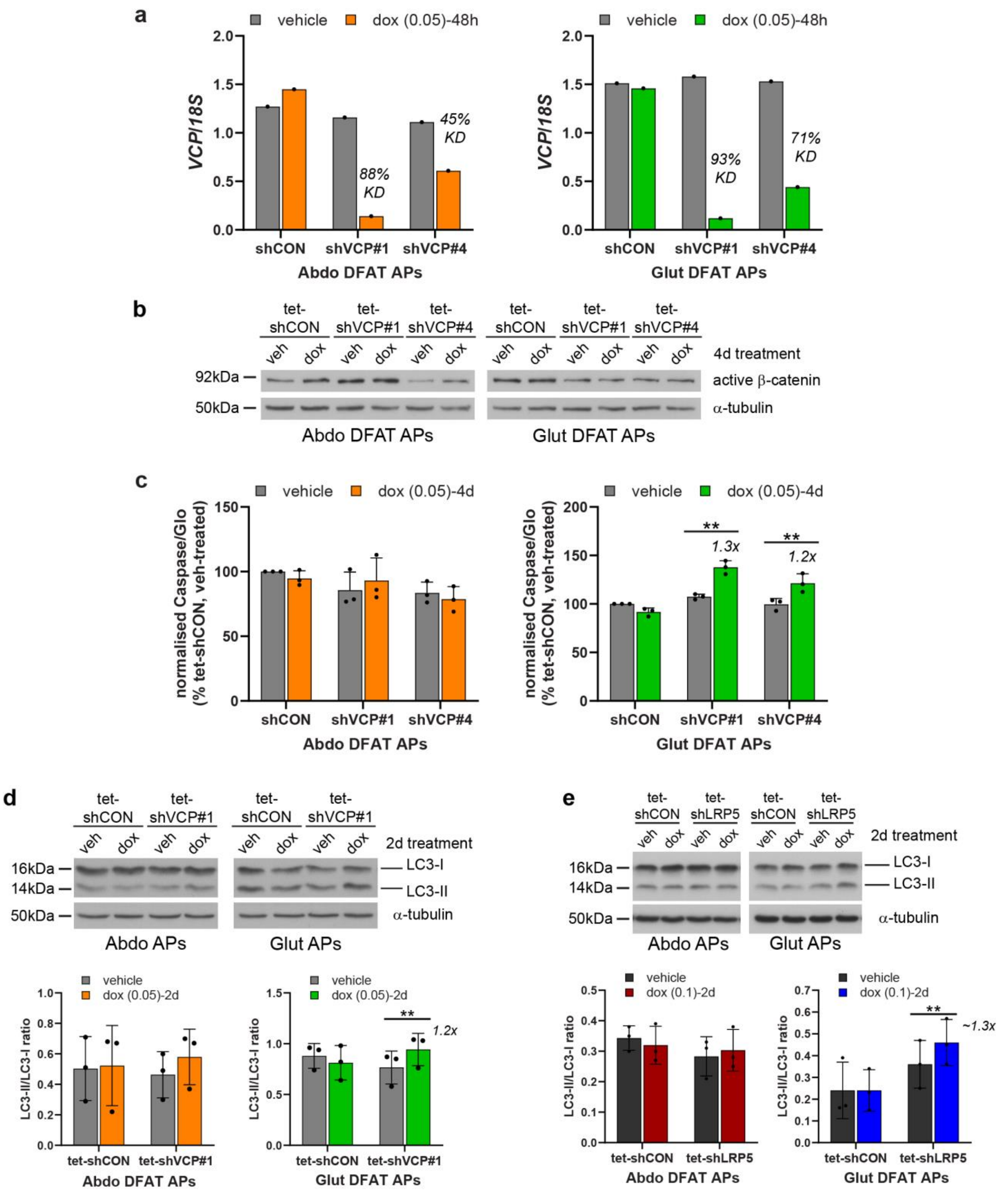

Supplementary Fig. 14. Effects of doxycycline-induced *VCP*-KD on DFAT AP biology. (**a**) qRT-PCR results showing *VCP*-KD by two independent shRNAs (shVCP#1 and shVCP#4)

following treatment with 0.05µg/ml doxycycline (dox) for 2 days. (**b**-**c**) Effects of 4-day doxycycline-induced *VCP*-KD in DFAT APs on (**b**) active β-catenin expression and (**c**) apoptosis (n = 3 independent experiments, results normalized to cell number; $(\text{genotype x dox})_{Abdo}$ $p = 0.02$; $(\text{genotype x dox})_{Glut}$ $p = 0.002$). Histograms are means + SD. (**d-e**) Effects of ~48h doxycycline-induced knockdown of (**d**) VCP and (**e**) LRP5 on autophagy in DFAT APs. Autophagy was evaluated in DFAT APs by Western blot and densitometry of the autophagic marker LC3-II. Histograms are means ± SD. Statistical tests: (**c**) Two-way RM ANOVA with Sidak's multiple comparisons test, (**d**, **e**) paired t-tests with two-stage step-up (Benjamini, Krieger, and Yekutieli)) correction for multiple testing (1% FDR). $^{**}p < 0.01$. α-tubulin was used as loading control for Western blots.

SUPPLEMENTARY TABLES

Supplementary Table 1. List of Taqman assays and antibodies.

Taqman assay	**Source**	**Assay ID**
18S	ThermoFisher Scientific	Hs99999901_s1
ADIPOQ	ThermoFisher Scientific	Hs00605917_m1
CDNK1A	ThermoFisher Scientific	Hs00355782_m1
CEBPA	ThermoFisher Scientific	Hs00269972_s1
CCL2 (MCP1)	ThermoFisher Scientific	Hs00234140_m1
DKK1	ThermoFisher Scientific	Hs00183740_m1
IL1A	ThermoFisher Scientific	Hs00380771_m1
IL6	ThermoFisher Scientific	Hs00985639_m1
INSR	ThermoFisher Scientific	Hs00961554_m1
IRS1	ThermoFisher Scientific	Hs00178563_m1
LIPE	ThermoFisher Scientific	Hs00193510_m1
LRP5	ThermoFisher Scientific	Hs00182031_m1
PLIN1	ThermoFisher Scientific	Hs00160173_m1
PNPLA2	ThermoFisher Scientific	Hs00386101_m1
PPARG2	ThermoFisher Scientific	Hs01115510_m1
SLC2A1	ThermoFisher Scientific	Hs00892681_m1
SLC2A3	ThermoFisher Scientific	Hs00359840_m1
SLC2A3 (EX2-3)	ThermoFisher Scientific	APMF26Y
SLC2A4	ThermoFisher Scientific	Hs00168966_m1
VCP	ThermoFisher Scientific	Hs00997642_m1
Antibody	**Source**	**Cat#**
Actin (I-19)-HRP goat pAb	Santa Cruz Biotechnology	sc-1616
cyclin D1 (M-20) rabbit pAb	Santa Cruz Biotechnology	sc-718
LRP5 (D80F2) rabbit mAb	Cell Signalling Technology	#5731
Active β-catenin (clone 8E7) mouse mAb	Millipore	#05-665
pAKT-S473 rabbit pAb	Cell Signalling Technology	#9271
total AKT rabbit pAb	Cell Signalling Technology	#9272
cMYC (E5Q6W) rabbit mAb	Cell Signalling Technology	#18583
α-tubulin rabbit pAb	Abcam	ab15246
Mono- and polyubiquitinylated conjugates recombinant monoclonal antibody (UBCJ2) (HRP conjugate)	Enzo	ENZ-ABS840HRP-0100
VCP mouse mAb	BioLegend Inc	#870902

Supplementary Table 2. GWAS summary statistics (sex-combined) used in Mendelian randomization study.

Trait	Source file	Database	PMID	Ancestry	Sample size	Consortium	% sample overlap with exposure
Heel eBMD	ebi-a-GCST006979	https://gwas.mrcieu.ac.uk/datasets/	30598549	European	426,824	UKB	
asatadjbmi3 [a]	0321_asatadjbmi3_bgen_stats.gz	https://cvd.hugeamp.org/datasets.html	35773277	Mixed	38,965	UKB	9%
gfatadjbmi3 [a]	0321_gfatadjbmi3_bgen_stats.gz	https://cvd.hugeamp.org/datasets.html	35773277	Mixed	38,965	UKB	9%
vatadjbmi3 [a]	0321_vatadjbmi3_bgen_stats.gz	https://cvd.hugeamp.org/datasets.html	35773277	Mixed	38,965	UKB	9%
BMI	bmi.giant-ukbb.meta-analysis.combined.23May2018.txt.gz (GCST009004)	https://www.ebi.ac.uk/gwas/	30239722	European	806,834	GIANT+UKB	53%
WHRadjBMI	whradjbmi.giant-ukbb.meta-analysis.combined.23May2018.txt.gz (GCST008999)	https://www.ebi.ac.uk/gwas/	30239722	European	694,649	GIANT+UKB	61%
Waist circumference (adjBMI)	ieu-a-67	https://gwas.mrcieu.ac.uk/datasets/	25673412	European	231,353	GIANT	
Hip circumference (adjBMI)	ieu-a-55	https://gwas.mrcieu.ac.uk/datasets/	25673412	European	211,114	GIANT	
Fasting glucose (adjBMI)	ebi-a-GCST90002232	https://gwas.mrcieu.ac.uk/datasets/	34059833	European	200,622	MAGIC	
Fasting insulin (adjBMI)	ebi-a-GCST90002238	https://gwas.mrcieu.ac.uk/datasets/	34059833	European	151,013	MAGIC	
logTAG_noUKB	without_UKB_logTG_INV_EUR_HRC_1KGP3_others_ALL.meta.singlevar.results.gz	http://csg.sph.umich.edu/willer/public/glgc-lipids2021/results/ancestry_specific/	34887591	European	864,240	GLGC	
HDL_noUKB	without_UKB_HDL_INV_EUR_HRC_1KGP3_others_ALL.meta.singlevar.results.gz	http://csg.sph.umich.edu/willer/public/glgc-lipids2021/results/ancestry_specific/	34887591	European	888,227	GLGC	
LDL_noUKB	without_UKB_LDL_INV_EUR_HRC_1KGP3_others_ALL.meta.singlevar.results.gz	http://csg.sph.umich.edu/willer/public/glgc-lipids2021/results/ancestry_specific/	34887591	European	842,660	GLGC	

[a] GWAS summary statistics were derived from a multi-ancestry study (>95% European participants).

Abbreviations: asatadjbmi3, BMI and height-adjusted abdominal subcutaneous adipose tissue; BMI, body mass index; eBMD, estimated bone mineral density; gfatadjbmi3, BMI and height-adjusted gluteofemoral adipose tissue; HDL, high-density lipoprotein; LDL, low-density lipoprotein; TAG, triglycerides; vatadjbmi3, BMI and height-adjusted visceral adipose tissue; WHRadjBMI, BMI-adjusted waist-to-hip ratio.

Supplementary Table 3. Clinical characteristics of *LRP5* gain-of-function (GoF) (A242T, N198S) cases and controls.

	***LRP5* GoF (n=6)**	**Controls (n=60)**	**p-value**
Sex	4F, 2M	40F, 20M	
Age (years)	43.0 ± 14.5	40.5 ± 7.4	0.69 †
Height (cm)	176.3 ± 11.2	170.2 ± 10.2	0.17
Weight (kg)	86.8 ± 19.6	80.4 ± 14.3	0.32
BMI (kg/m^2)	27.7 ± 4.3	27.8 ± 4.2	0.97
**DXA measurements**			
Fat android (kg) *	2.2 (2.0, 2.7)	2.2 (1.9, 2.9)	0.96
Fat gynoid (kg) *	5.2 (4.6, 5.8)	4.8 (3.6, 5.5)	0.55
Fat visceral (kg) *	0.61 (0.40, 0.75)	0.64 (0.42, 1.12)	0.82
Fat legs (kg) *	10.9 (10.6, 12.4)	9.1 (7.3, 10.8)	0.17
Total fat mass (kg) *	30.5 (28.9, 31.8)	27.3 (23.3, 30.2)	0.21
Total fat percentage *	38.2 (29.3, 40.8)	37.7 (31.1, 40.4)	0.97
Android/leg fat ratio *	0.20 (0.16, 0.22)	0.24 (0.20, 0.30)	**0.037**
Total lean mass (kg) *	50.1 (41.2, 52.7)	45.8 (40.4, 54.9)	0.67
Lean mass legs (kg) *	16.3 (13.4, 20.2)	15.7 (14.0, 18.9)	0.87
Total BMD[a] (g/cm^2) *	2.0 (1.8, 2.1)	1.2 (1.1, 1.3)	**2.20×10^{-8}**
**Biochemistry**			
Fasting glucose (mmol/L)	4.9 ± 0.4	5.3 ± 0.5	0.052
Fasting insulin (mIU/L) *	9.9 (5.1, 11.9)	14.4 (9.6, 18.4)	**0.041**
HOMA-IR *	2.1 (1.0, 2.9)	3.3 (2.2, 4.6)	**0.027**
HOMA-B *	129 (110, 150)	161 (128, 204)	0.13
NEFA (µmol/l) *	385 (301, 543)	485 (287, 618)	0.48
Adipo-IR*	20 (17, 26)	36 (22, 63)	0.068
Triglycerides (mmol/L) *	0.7 (0.6, 1.2)	1.0 (0.8, 1.4)	0.21

Data presented as mean ± SD and *median (interquartile range) for skewed variables. P-values obtained from two-tailed Student's t-test and *Mann-Whitney test as applicable. †with Welch's correction for unequal variance.

Abbreviations: Adipo-IR, adipose-tissue insulin resistance; BMD, bone mineral density; BMI, body mass index; DXA, dual-energy X-ray absorptiometry; HOMA-IR, Homeostatic Model Assessment for Insulin Resistance; HOMA-B, Homeostatic Model Assessment for Insulin Secretion; NEFA, non-esterified fatty acids.

Supplementary Table 4. Biochemical and body composition profile of individuals with *LRP5*-gain of function variants within cluster of age, sex and BMI-matched controls.

	Cases							Controls					
	**C1**	**C2**	**C3***	**C4**	**C5**	**C6**	**Pooled cases mean (95%CI)**	**#1**	**#2**	**#3**	**#4**	**#5**	**#6**
Sex	F	F	F	F	M	M		10F	10F	10F	10F	10M	10M
Z-Age	0.00	-0.07	2.99	-0.19	-2.52	0.06		0.00 (-1.58, 1.58)	0.01 (-1.52, 1.39)	-0.30 (-0.44, -0.09)	0.02 (-1.76, 0.86)	0.25 (-0.33, 1.13)	-0.01 (-1.16, 1.27)
Z-BMI	0.08	0.03	0.10	-0.51	0.23	-0.35		-0.01 (-1.47, 1.08)	0.00 (-1.82, 1.25)	-0.01 (-1.48, 1.62)	0.05 (-1.84, 1.28)	-0.02 (-2.07, 1.18)	0.03 (-0.97, 1.88)
Z-height	-0.15	2.44	0.75	0.75	-1.02	2.09	**0.81 (-0.56, 2.19)**	0.01 (-1.49, 1.49)	-0.24 (-1.18, 1.11)	-0.07 (-1.57, 1.33)	-0.08 (-1.72, 0.97)	0.10 (-1.38, 1.88)	0.21 (-1.33, 1.13)
Z-weight	-0.17	2.46	0.76	0.62	-1.04	2.16	**0.80 (-0.61, 2.21)**	0.02 (-1.55, 1.56)	-0.25 (-1.28, 1.05)	-0.08 (-1.49, 1.40)	-0.06 (-1.58, 1.23)	0.10 (-1.56, 1.61)	-0.22 (-1.26, 1.12)
Z-BMI	0.08	0.03	0.10	-0.51	0.23	-0.35	**-0.07 (-0.38, 0.24)**	-0.01 (-1.47, 1.08)	0.00 (-1.82, 1.25)	-0.01 (-1.48, 1.62)	0.05 (-1.84, 1.28)	-0.02 (-2.07, 1.18)	0.03 (-0.97, 1.88)
Biochemistry													
Z-glucose (fasting)	0.07	-1.28	0.14	-1.44	-1.47	-1.23	**-0.87 (-1.66, -0.07)**	-0.01 (-1.46, 1.91)	0.13 (-1.09, 1.84)	-0.01 (-1.49, 2.34)	0.14 (-1.83, 1.45)	0.15 (-1.35, 1.61)	0.12 (-1.13, 1.73)
Z-insulin (fasting)	-0.29	-1.38	-0.14	-0.98	-1.22	-0.98	**-0.83 (-1.36, -0.30)**	0.03 (-2.14, 1.10)	0.14 (-1.47, 2.09)	0.01 (-1.97, 1.55)	0.10 (-1.01, 1.83)	0.12 (-1.36, 1.78)	0.10 (-1.33, 1.88)
Z-HOMA-IR	-0.29	-1.28	-0.13	-1.05	-1.30	-0.99	**-0.84 (-1.37, -0.31)**	0.03 (-2.00, 1.26)	0.13 (-1.33, 2.23)	0.01 (-1.73, 1.73)	0.10 (-1.05, 1.67)	0.13 (-1.28, 1.61)	0.10 (-1.19, 2.02)
Z-HOMA-B	-0.34	-1.28	-0.34	-0.60	-0.35	-0.55	**-0.58 (-0.96, -0.19)**	0.03 (-1.81, 1.92)	0.13 (-1.72, 1.89)	0.03 (-2.16, 1.94)	0.06 (-1.13, 2.25)	0.03 (-1.52, 1.89)	0.06 (-1.65, 1.61)
Z-NEFA	-0.90	1.06	0.15	-0.85	-0.45	-0.33	**-0.22 (-0.99, 0.55)**	0.09 (-1.56, 1.57)	-0.11 (-1.48, 1.53)	0.02 (-1.73, 1.23)	0.08 (-1.48, 1.40)	0.04 (-0.61, 2.82)	0.03 (-1.63, 1.72)
Z-Adipo-IR	-0.77	-0.98	-0.03	-1.16	-0.75	-0.74	**-0.74 (-1.14, -0.33)**	0.08 (-1.29, 1.83)	0.10 (-1.48, 2.19)	0.00 (-1.40, 1.90)	0.12 (-1.12, 1.50)	0.07 (-0.68, 2.83)	0.07 (-1.21, 2.17)
Z-triglycerides	-0.84	-0.47	-0.93	-0.45	-0.67	0.20	**-0.53 (-0.95, -0.10)**	0.08 (-1.15, 2.07)	0.05 (-0.83, 2.64)	0.09 (-1.05, 2.14)	0.04 (-1.07, 2.67)	0.07 (-1.31, 2.18)	-0.02 (-1.56, 1,41)
DXA measurements													
Z-Fat android	-0.93	-0.09	0.08	-0.42	-1.57	0.23	**-0.45 (-1.17, 0.27)**	0.09 (-1.10, 1.83)	0.01 (-0.99, 2.15)	-0.01 (-0.97, 2.77)	0.04 (-0.83, 2.01)	0.16 (-0.87, 2.40)	-0.02 (-2.60, 1.33)
Z-Fat gynoid	-1.01	0.76	0.72	0.90	-1.41	1.29	**0.21 (-0.97, 1.39)**	0.10 (-1.37, 1.97)	-0.08 (-1.27, 1.53)	-0.07 (-1.77, 1.38)	-0.09 (-1.38, 1.41)	0.14 (-1.13, 1.52)	-0.13 (-2.15, 1.37)
Z-Fat visceral	-0.44	-1.09	0.28	0.17	0.06	-0.62	**-0.27 (-0.84, 0.27)**	0.04 (-1.22, 2.15)	0.11 (-1.09, 1.58)	-0.03 (-1.80, 1.98)	-0.02 (-1.91, 1.57)	-0.01 (-1.23, 2.49)	0.06 (-1.27, 1.84)
Z-Fat legs	0.53	1.23	1.05	0.92	-0.69	1.83	**0.81 (-0.08, 1.70)**	-0.05 (-1.41, 2.16)	-0.12 (-1.42, 1.74)	-0.11 (-1.58, 1.19)	-0.09 (-1.42, 1.58)	0.07 (-1.14, 1.58)	-0.18 (-1.94, 1.17)
Z-Total fat mass	0.17	1.44	1.09	0.23	-1.46	1.00	**0.41 (-0.69, 1.51)**	-0.02 (-1.78, 1.49)	-0.14 (-1.30, 1.30)	-0.11 (-1.86, 1.98)	-0.02 (-1.80, 1.26)	0.15 (-1.07, 1.59)	-0.10 (-2.62, 1.23)
Z-Total fat percentage	0.31	-0.01	0.63	-0.95	-1.32	0.06	**-0.21 (-1.01, 0.58)**	-0.03 (-0.87, 2.53)	0.00 (-1.29, 1.80)	-0.06 (-1.66, 1.72)	0.09 (-1.95, 1.88)	0.13 (-1.21, 1.95)	-0.01 (-2.84, 0.66)
Z-Android/leg fat ratio	-1.15	-0.92	-0.43	-1.01	-1.21	-1.00	**-0.95 (-1.24, -0.66)**	0.11 (-1.26, 2.12)	0.09 (-1.22, 2.37)	0.04 (-0.95, 2.47)	0.10 (-1.43, 1.27)	0.12 (-0.94, 2.22)	0.10 (-1.84, 1.64)
Z-Total lean mass	-0.66	1.64	0.00	0.57	-0.55	0.84	**0.31 (-0.62, 1.24)**	0.07 (-2.08, 1.43)	-0.16 (-1.44, 1.61)	0.00 (-1.14, 1.89)	-0.06 (-1.51, 1.22)	0.05 (-1.77, 1.60)	-0.08 (-1.11, 2.37)
Z-Lean legs	-1.19	0.99	-0.36	0.81	-0.58	1.00	**0.11 (-0.88, 1.10)**	0.12 (-1.62, 1.30)	-0.10 (-1.39, 1.77)	0.04 (-1.69, 1.63)	0.08 (-1.59, 1.27)	-0.06 (-1.59, 1.67)	-0.10 (-1.46, 1.52)
Z-Total BMD	2.77	2.96	2.77	2.72	2.82	2.94	**2.83 (2.73, 2.93)**	-0.28 (-0.67, 0.57)	-0.30 (-0.61, 0.06)	-0.28 (-1.01, 0.37)	-0.27 (-0.84, 0.55)	-0.28 (-0.73, 0.62)	-0.29 (-0.73, 0.06)

Data represents SD change in outcome for each case within the cluster of age and gender matched controls.

Z-scores for controls data are presented as means (min, max).

*C3 was matched with ten 48-50 year-old sex and BMI-matched controls (median age = 49 years).

Abbreviations: Adipo-IR, adipose-tissue insulin resistance; BMD, bone mineral density; BMI, body mass index; DXA, dual-energy X-ray absorptiometry; HOMA-IR, Homeostatic Model Assessment for Insulin Resistance; HOMA-B, Homeostatic Model Assessment for Insulin Secretion; NEFA, non-esterified fatty acids.

Supplementary Table 5. Clinical characteristics of *LRP5* loss-of-function (LoF) (V667M) cases and controls.

	***LRP5* LoF (n = 23)**	**Controls (n = 229)**	**p-value**
Sex	15F, 8M	150F, 79M	
Age (years)	42.5 ± 6.7	41.8 ± 5.2	0.56
Height (cm)	167.2 ± 10.2	170.7 ± 8.8	0.08
Weight (kg)	72.6 ± 18.5	75.0 ± 15.4	0.49
BMI (kg/m^2)	25.6 ± 4.1	25.6 ± 4.0	0.98
**DXA measurements***			
Fat android (kg) †	1.8 (1.2, 3.1)	1.9 (1.2, 2.7)	0.96
Fat gynoid (kg) †	3.5 (3.1, 4.5)	4.1 (3.4, 5.0)	0.17
Fat visceral (kg) †	0.46 (0.16, 1.52)	0.43 (0.15, 1.19)	0.84
Fat legs (kg) †	6.7 (5.7, 8.6)	8.0 (6.5, 9.7)	0.14
Total fat mass (kg) †	21.2 (17.8, 32.8)	23.8 (19.5, 29.1)	0.69
Total fat percentage †	31.8 (29.6, 35.7)	33.0 (28.4, 37.6)	0.75
Android/leg fat ratio †	0.24 (0.15, 0.42)	0.22 (0.16, 0.31)	0.46
Total lean mass (kg) †	43.7 (38.3, 55.0)	43.9 (39.8, 51.4)	0.87
Lean mass legs (kg) †	15.6 (12.3, 20.3)	15.2 (13.3, 17.6)	0.95
Total BMD[a] (g/cm^2) †	1.2 (1.1, 1.2)	1.2 (1.1, 1.3)	**0.026**
**Biochemistry**			
Fasting glucose (mmol/L)	5.1 ± 0.5	5.2 ± 0.4	0.75
Fasting insulin (mIU/L) †	12.5 (9.0, 18.8)	11.2 (8.1, 14.5)	0.22
HOMA-IR †	3.0 (2.0, 4.3)	2.5 (1.8, 3.2)	0.3
HOMA-B †	156 (127, 211)	137 (105, 171)	0.08
NEFA (µmol/l) †	537 (346, 757)	465 (321, 608)	0.4
Adipo-IR †	36 (25, 51)	29 (20, 45)	0.16
Triglycerides (mmol/L) †	1.0 (0.8, 1.7)	0.9 (0.6, 1.3)	0.08

Data presented as mean ± SD and †median (interquartile range) for skewed variables. P-values obtained from two-tailed Student's t-test and †Mann-Whitney test as applicable.
*DXA measurements available on 14 cases (10F, 4M) and their matched 139 controls.
Abbreviations: Adipo-IR, adipose-tissue insulin resistance; BMD, bone mineral density; BMI, body mass index; DXA, dual-energy X-ray absorptiometry; HOMA-IR, Homeostatic Model Assessment for Insulin Resistance; HOMA-B, Homeostatic Model Assessment for Insulin Secretion; NEFA, non-esterified fatty acids.

Supplementary Table 6. Clinical characteristics of subjects undergoing OGTTs.

	***LRP5* GoF (n = 6)**	**Controls (n = 10)**	**P-value**
	4F, 2M	6F, 4M	
Age (years)	43.0 ± 14.5	45.9 ± 9.8	0.64
Height (cm)	176.3 ± 11.2	173.4 ± 8.1	0.55
Weight (kg)	86.8 ± 19.6	82.2 ± 10.2	0.61
BMI (kg/m^2)	27.7 ± 4.3	27.4 ± 3.5	0.87
**DXA Measurements**			
Fat android (kg) *	2.2 (2.0, 2.7)	2.5 (1.7, 3.1)	0.79
Fat legs (kg) *	10.9 (10.6, 12.4)	10.1 (7.6, 13.1)	0.64
Total fat mass (kg) *	30.5 (28.9, 31.8)	29.3 (27.6, 30.5)	0.49
Total fat percentage *	38.2 (29.3, 40.8)	35.5 (31.8, 41.1)	1
Android/leg fat ratio *	0.20 (0.16, 0.22)	0.26 (0.20, 0.35)	0.06
Total lean mass (kg) *	50.1 (41.2, 52.7)	52.5 (45.3, 56.4)	0.64
Lean mass legs (kg) *	16.3 (13.4, 20.2)	18.2 (15.0, 20.0)	0.87
Total BMD (g/cm^2) *	2.0 (1.8, 2.1)	1.3 (1.2, 1.3)	**2.50E-04**
**Biochemistry**			
Fasting glucose (mmol/L)	4.9 ± 0.4	5.3 ± 0.3	**0.03**
Glucose - 30min (mmol/L)	7.5 ± 0.6	8.1 ± 1.3	0.27
Glucose - 60min (mmol/L)	6.6 ± 1.8	8.1 ± 2.0	0.16
Glucose - 90min (mmol/L)	6.2 ± 1.4	7.4 ± 1.8	0.19
Glucose - 120min (mmol/L)	5.7 ± 1.1	6.8 ± 1.5	0.15
AUC-glucose *	752 (688, 866)	878 (797, 973)	0.07
Fasting insulin (mIU/L) *	9.9 (5.1, 11.9)	9.5 (6.5, 10.6)	0.71
Insulin - 30min (mIU/L) *	52.5 (42.5, 63.4)	40.8 (35.6, 106.6)	0.49
Insulin - 60min (mIU/L) *	51.0 (35.7, 69.4)	44.8 (41.7, 98.1)	0.87
Insulin - 90min (mIU/L) *	49.2 (33.5, 68.1)	61.1 (27.8, 91.3)	0.56
Insulin - 120min (mIU/L) *	25.1 (21.8, 65.4)	42.2 (31.9, 86.3)	0.22
AUC-insulin *	5284 (4189, 7032)	5249 (4280, 9709)	0.87
HOMA-IR *	2.1 (1.0, 2.9)	2.2 (1.6, 2.6)	0.64
HOMA-B *	129 (110, 150)	106 (86, 121)	0.26
Matsuda Index *	86.6 (68.0, 136.1)	77.5 (52.2, 117.2)	0.43
NEFA 0min (µmol/l) *	385 (301, 543)	475 (385, 597)	0.26
NEFA 30min (µmol/l) *	223 (70, 312)	463 (357, 673)	**0.003**
NEFA 60min (µmol/l) *	63 (51, 65)	164 (96, 245)	**0.003**
NEFA 90min (µmol/l) *	19 (10, 42)	109 (33, 170)	**0.02**
NEFA 120min (µmol/l) *	13 (10, 34)	30 (15, 87)	0.18
AUC-NEFA *	15743 (11938, 19919)	31918 (22407, 37655)	**0.001**
Adipo-IR *	20 (17, 26)	27 (23, 36)	0.22
Cholesterol (mmol/L)	4.9 ± 0.5	5.0 ± 0.7	0.64
Triglyceride (mmol/L) *	0.72 (0.62, 1.22)	0.84 (0.67, 1.23)	0.64
HDL-cholesterol (mmol/L) *	1.5 (1.0, 1.7)	1.4 (1.2, 1.6)	0.96

Data presented as mean ± SD and *median (interquartile range) for skewed variables.
P-values obtained from two-tailed Student's t-test and *Mann-Whitney test as applicable.
Abbreviations: Adipo-IR, adipose tissue insulin resistance; BMD, bone mineral density; BMI, body mass index; DXA, dual-energy X-ray absorptiometry; HDL, high density lipoprotein; HOMA-IR, Homeostatic Model Assessment for Insulin Resistance; HOMA-B, Homeostatic Model Assessment for Insulin Secretion; NEFA, non-esterified fatty acids; OGTT, oral glucose tolerance test.

Supplementary Table 7. Anthropometric characteristics of subjects from whom DXA scans and paired abdominal and gluteal AT biopsies were obtained for current gene expression studies.

	**All**	**With DXA**
n	43F, 37M	43F
Age (years)*	47.5 ± 1.0 (31.8, 67.4)	50.7 ± 1.2 (37.5, 67.4)
BMI (kg/m^2) *	26.9 ± 0.5 (20.1, 48.9)	27.4 ± 0.6 (22.1, 35.3)

*mean ± SEM (min, max). Abbreviations: AT, adipose tissue; DXA, dual-energy X-ray absorptiometry; BMI, body mass index.

Supplementary Table 8. Mendelian randomization estimates of the effects of heel eBMD (g/cm^2) on anthropometric, blood pressure and metabolic traits.

Primary analyses			IVW			Egger				Weighted-median		
**Exposure**	**Outcome**	***N* SNP**	**beta**	**se**	**p-value**	**beta**	**se**	**p-value**	**Intercept p-value**	**beta**	**se**	**p-value**
heel eBMD	asatadjbmi3	501	-0.012	0.017	0.49	-0.008	0.030	0.79	0.87	-0.010	0.024	0.69
	gfatadjbmi3	501	-0.102	0.020	**6.41×10^{-7}**	-0.089	0.035	**0.013**	0.65	-0.072	0.027	**0.0072**
	vatadjbmi3	501	0.009	0.020	0.65	0.016	0.034	0.65	0.81	-0.028	0.026	0.28
	BMI	498	0.015	0.009	0.077	0.003	0.015	0.82	0.33	0.019	0.006	**0.0036**
	WHRadjBMI	498	0.007	0.011	0.54	0.007	0.018	0.69	0.96	-0.011	0.007	0.15
	Waist circumference (adjBMI)	368	-0.019	0.015	0.20	-0.012	0.027	0.67	0.72	-0.043	0.017	**0.013**
	Hip circumference (adjBMI)	368	-0.048	0.018	**0.0084**	-0.015	0.032	0.64	0.21	-0.009	0.017	0.61
	Fasting glucose (adjBMI) || id:ebi-a-GCST90002232	497	0.004	0.005	0.45	0.009	0.010	0.34	0.52	0.002	0.007	0.76
	Fasting insulin (adjBMI) || id:ebi-a-GCST90002238	499	0.014	0.006	**0.011**	0.019	0.010	**0.048**	0.51	-0.003	0.008	0.73
	logTAG_noUKB	500	0.012	0.009	0.15	-0.010	0.015	0.50	0.067	-0.002	0.006	0.72
	HDL_noUKB	499	-0.019	0.010	0.050	-0.016	0.017	0.34	0.85	0.004	0.007	0.55
	LDL_noUKB	499	-0.012	0.013	0.36	0.004	0.022	0.84	0.37	-0.006	0.006	0.37
heel eBMD_maf≥0.05 [a]	asatadjbmi3	457	-0.009	0.018	0.63	-0.002	0.032	0.96	0.79	-0.009	0.026	0.73
	gfatadjbmi3	457	-0.107	0.022	**7.07×10^{-7}**	-0.097	0.038	**0.012**	0.76	-0.063	0.028	**0.027**
	vatadjbmi3	457	0.010	0.021	0.64	0.019	0.037	0.60	0.75	-0.036	0.026	0.17
	BMI	455	0.015	0.009	0.099	0.001	0.016	0.93	0.30	0.019	0.007	**0.0046**
	WHRadjBMI	455	0.008	0.011	0.50	0.009	0.020	0.67	0.96	-0.010	0.008	0.17
	Waist circumference (adjBMI)	360	-0.020	0.016	0.20	-0.012	0.027	0.66	0.74	-0.044	0.017	**0.010**
	Hip circumference (adjBMI)	360	-0.050	0.018	**0.0067**	-0.019	0.032	0.55	0.25	-0.011	0.018	0.54
	Fasting glucose (adjBMI) || id:ebi-a-GCST90002232	454	0.003	0.006	0.63	0.007	0.010	0.50	0.63	0.002	0.007	0.78
	Fasting insulin (adjBMI) || id:ebi-a-GCST90002238	456	0.016	0.006	**0.0072**	0.024	0.010	**0.023**	0.35	-0.002	0.008	0.81
	logTAG_noUKB	456	0.014	0.009	0.12	-0.008	0.016	0.61	0.095	-0.002	0.006	0.78
	HDL_noUKB	455	-0.018	0.010	0.066	-0.015	0.018	0.40	0.81	0.004	0.007	0.56
	LDL_noUKB	455	-0.011	0.014	0.42	0.007	0.024	0.76	0.36	-0.007	0.007	0.32

[a] genetic instruments with MAF ≥0.05.

Abbreviations: asatadjbmi3, BMI and height-adjusted abdominal subcutaneous adipose tissue; BMI, body mass index; eBMD, estimated bone mineral density; gfatadjbmi3, BMI and height-adjusted gluteofemoral adipose tissue; HDL, high-density lipoprotein; LDL, low-density lipoprotein; TAG, triglycerides; vatadjbmi3, BMI and height-adjusted visceral adipose tissue; WHRadjBMI, BMI-adjusted waist-to-hip ratio.

Highlighted are results with $p < 0.05$ in IVW and at least one other sensitivity analysis.

Supplementary Table 9. Partial correlations (Spearman's) of measurements of body-fat distribution (DXA) with *VCP* mRNA levels from abdominal and gluteal adipose tissue fractions from 43 women, adjusted for age and % total fat mass.

	**SC Abdominal APs**		**Gluteal APs**		**Abdo ADS**		**Glut ADS**	
**Traits**	***rho***	***P***	***rho***	***P***	***rho***	***P***	***rho***	***P***
Android fat mass (g)	-0.067	0.57	-0.049	0.68	0.263	**0.022**	0.059	0.61
Gynoid fat mass (g)	0.093	0.43	0.174	0.141	0.197	0.088	0.103	0.38
Leg fat mass (g)	0.204	0.082	0.281	**0.016**	0.109	0.35	0.136	0.25
Android/Gynoid fat ratio	-0.177	0.13	-0.296	**0.011**	0.021	0.86	0.025	0.83
Android/Leg fat ratio	-0.228	0.051	-0.347	**0.003**	0.089	0.45	0.008	0.95

Abbreviations: DXA, dual-energy X-ray absorptiometry; SC, subcutaneous; APs, adipose progenitors; ADS, adipocytes.

Supplementary Table 10. Comparison of body composition (DXA) of women with *LRP5* gain-of-function (GoF) (A242T, N198S) variants and sex, age, BMI-matched controls.

	***LRP5* GoF** **Mean difference (95% confidence interval)**	**p-value**
Sex, case (age-matched controls)	4F (48F)	
Age (years)‡	0.0 ± 4.8	1
BMI (kg/m^2)‡	0.2 ± 1.9	0.93
DXA measurements		
Z-Fat android	-0.44 (-1.46, 0.57)	0.39
Z-Fat gynoid	0.24 (-0.78, 1.26)	0.64
Z-Fat visceral	-0.42 (-1.44, 0.59)	0.22
Z-Fat legs	0.82 (-0.18, 1.82)	0.10
Z-Total fat mass	0.62 (-0.39, 1.62)	0.23
Z-total fat percentage	-0.13 (-1.16, 0.89)	0.79
Z-Android/leg fat ratio	-1.75 (-2.64, -0.85)	**3×10^{-4}**
Z-Total lean mass	0.44 (-0.57, 1.46)	0.39
Z-Lean legs	0.08 (-0.94, 1.11)	0.87
Z-Total BMD	3.14 (2.75, 3.53)	**3×10^{-20}**

Data represent mean difference (case-controls) in outcome (Z-transformed) for each individual within the cluster of sex, age, and BMI-matched controls.
C3, the 68-year-old *LRP5* GoF variant carrier (BMI = 27.3 kg/m^2) was matched with eighteen women age [median (min, max) =59 (55, 67) years] and BMI [median (min, max) = 27.3 (26.1, 28.5) kg/m^2], for whom DEXA was available. P-values were obtained from two-sample t-tests.
‡mean (SD) of the case from its controls within each cluster.
Abbreviations: BMD, bone mineral density; BMI, body mass index, DXA, dual-energy X-ray absorptiometry.

Supplementary Table 11. Comparison of body composition (DXA) of women with *LRP5* gain-of-function (GoF) (A242T, N198S) variants and matched controls within a cluster of sex- and BMI-matched individuals 5-20 years younger.

	***LRP5* GoF** **Mean difference (95% confidence interval)**	**p-value**	**controls** **Mean difference (95% confidence interval)**	**p-value**
Sex, case/control (younger controls)	4F (40F)		48F (40F)	
Age (years)‡	11.2 ± 3.9	**0.006**	11.2 ± 1.7	**3×10^{-9}**
BMI (kg/m^2)‡	0.0 ± 1.9	0.98	-0.1 ± 0.8	0.89
DXA measurements				
Z-Fat android	-0.08 (-1.11, 0.95)	0.88	0.28 (-0.13, 0.70)	0.18
Z-Fat gynoid	0.39 (-0.63, 1.42)	0.44	0.09 (-0.33, 0.51)	0.66
Z-Fat visceral	0.35 (0.01, 0.69)	**0.045**	0.65 (0.25, 1.05)	**0.002**
Z-Fat legs	0.84 (-0.16, 1.84)	0.098	0.08 (-0.34, 0.50)	0.71
Z-Total fat mass	0.79 (-0.21, 1.79)	0.12	0.17 (-0.25, 0.59)	0.41
Z-total fat percentage	0.33 (-0.70, 1.36)	0.52	0.40 (-0.01, 0.81)	0.057
Z-Android/leg fat ratio	-0.69 (-1.03, -0.34)	**3×10^{-4} ***	0.66 (0.25, 1.07)	**0.002***
Z-Total lean mass	0.22 (-0.81, 1.25)	0.67	-0.27 (-0.68, 0.15)	0.21
Z-Lean legs	-0.26 (-1.29, 0.77)	0.62	-0.38 (-0.79, 0.03)	0.071
Z-Total BMD	3.00 (2.82, 3.17)	**4×10^{-19} ***	-0.59 (-1.01, -0.18)	**0.005**

Data represent mean difference [(case-younger controls) or (controls - younger controls)] in outcome (Z-transformed) for each individual within the cluster of sex and BMI-matched controls who are: 9-11 years younger for *LRP5* GoF carriers C1 & C2, 18-20 years younger for C3, and 5-7 years younger for C4.

P-values were obtained from two-sample t-tests (*with Welch correction).

‡mean (SD) of the case from its controls within each cluster.

Abbreviations: BMD, bone mineral density; BMI, body mass index, DXA, dual-energy X-ray absorptiometry.

SUPPLEMENTARY METHODS

Human participants:

The Oxford Biobank (OBB) [1] comprises a randomized, age-stratified sample obtained from Oxfordshire and the Thames Valley in the UK. The Thames Valley Primary Care Agency has enabled random recruitment by providing lists of Oxfordshire residents registered with a local general practitioner and aged 30–50 years. Individuals with a previous diagnosis of myocardial infarction or heart failure currently on treatment; untreated malignancy; other ongoing systemic diseases, and pregnant women were excluded from participation. The OBB recruitment began in 1999 and included 7640 individuals (4316 women and 3324 men) as of October 2016. OBB subjects were genotyped on the Illumina Human Exome BeadChip and Affymetrix UK Biobank Axiom arrays. Genotype imputation was performed using the Affymetrix UK Biobank Axiom array with Haplotype Reference Consortium (HRC), 1000Genome and UK10K reference panels using IMPUTE2 software. Whole body DXA was performed using a Lunar iDXA scanner (GE Healthcare, Little Chalfont, U.K.) and the acquired images processed using the enCORE v14.1 software.

The High Bone Mass (HBM) study is a UK-based multi-centered observational study of adults with unexplained HBM identified by screening 335,115 DXA scans from 13 UK DXA databases from which 258 HBM probands with BMD Z-score ≥+3.2 were identified. All participants were clinically assessed by one doctor using a standardized structured history and examination questionnaire, after which total-body Lunar prodigy DXA scans were performed [2]. Written informed consent was collected for all, in line with the Declaration of Helsinki. Participants were excluded if they were aged <18 years, pregnant, or unable to provide written informed consent for any reason. This study was approved by the Bath Multicenter Research Ethics Committee (REC) and at each NHS Local REC. Cases with HBM *LRP5* mutations were identified by targeted sequencing of exons 2-4 of *LRP5* (i.e. the sites of previously described HBM cases).

An invitation letter along with the study information and response sheet were sent to all participants. Individuals who expressed willingness to enroll in this study were contacted by telephone or email, in order to convey a brief overview of the study aims and objectives, by trained research nurses. All studies were approved by the Oxfordshire Clinical Research Ethics Committee and all volunteers gave written, informed consent (IRAS Ethics approval 18/SC/0588).

Plasma biochemistry: Plasma chemistry was performed on an ILAB 650 clinical analyser (Instrumentation Laboratory UK) [1]. Homeostatic Model Assessment for Insulin Resistance (HOMA-IR), HOMA of β-cell function (HOMA-B), AT insulin resistance (Adipo-IR), and Matsuda Index were calculated as follows: HOMA-IR = fasting glucose (mmol/L) × fasting insulin (mIU/L)/22.5; HOMA-B = [20 × fasting insulin (mIU/L)]/[glucose (mmol/L) - 3.5]; Adipo-IR = fasting NEFA (mmol/L) × fasting insulin (pmol/L); Matsuda Index = 10,000/√(fasting glucose x fasting insulin x AUC-glucose/120 x AUC-insulin/120).

Cell culture and *in vitro* adipogenesis: DFAT cells were maintained and plated under tetracycline-free conditions in DMEM-F12 supplemented with 10% FBS (ThermoFisher Scientific Gibco, #26140079), 2mM L-glutamine, 0.25ng/ml fibroblast growth factor, 100 units/ml penicillin and 100µg/ml streptomycin (and 2µg/ml puromycin or 20µg/ml hygromycin B, as appropriate). For differentiation of APs to adipocytes, confluent cells were cultured for 14 days in a standard adipogenic medium (DMEM-F12 containing 2mM L-glutamine, 100 units/ml penicillin, 100µg/ml streptomycin, 17µM pantothenate, 100nM human insulin, 10nM 3,3',5-triiodo-L-thyronine, 33µM biotin, 10µg/ml human transferrin and 1µM dexamethasone). For the first 4 days, 250µM 3-isobutyl-1-methylxanthine and 4µM troglitazone were added to the adipogenic medium.

WNT surrogate: We investigated the effects of a WNT surrogate-Fc fusion recombinant protein (ThermoFisher Scientific, PHG0401) [3,4] (WNTsurr) on proliferation and differentiation in abdominal and gluteal control and LRP5 knockdown APs. This surrogate consists of the LRP-binding domain of DKK1 linked to a selective, high-affinity binder that interacts with FZD1, FZD2, FZD5, FZD7, and FZD8, and has been shown to recapitulate the WNT3a global transcriptional response in human iPSCs [5]. All five receptors, as well as *FZD4* and *FZD6* (which are not engaged by the WNT surrogate), are expressed in abdominal and gluteal DFAT APs, with *FZD1* and *FZD7* expression being the highest (our unpublished data). To determine the dose of WNTsurr to use in our experiments, APs transduced with 7TFP were treated with a range of WNTsurr concentrations (3.9-1000pM) and compared with that of CHIR99021 (**Supplementary Fig. 11a-b**). To assess its effects on proliferation and adipogenesis, 25pM or 100 pM WNTsurr, or vehicle, were added to the growth or adipogenic media, respectively, with or without doxycycline.

Mitotic timing: Cell synchronization and life cell imaging: For imaging, cells were plated into 6-well glass-bottom plates (Cellvis, #P06-1.5H-N) at 50,000 cells/well 72 hours prior to the start of imaging. In parallel, cells were plated for Western blot. The next day, cells were treated with 0.1µg/ml doxycycline (or vehicle) for 24 hours, then subjected to a 17-hour 2mM thymidine block to arrest cells in S-phase. To release cells from the thymidine block, cells were washed with 4 x 3ml pre-warmed PBS and 1 x 3ml pre-warmed media. After the final wash, cells were cultured 1ml of pre-warmed media with 0.1µg/ml doxycycline or vehicle control, as appropriate.

Six hours after the thymidine washout 50nM SiR-DNA (Spirochrome, #SC007) was added to stain DNA. Imaging began 7 hours after the thymidine release (48 hours after depletion). Imaging was performed using a 20x/0.75 NA air objective on an EVOS M7000 (ThermoFisher) microscope with software version 2.0.2094.0, equipped with an onstage incubator and DAPI, GFP, Texas Red, and Cy5 light cubes. Cells were imaged at 37°C under 5% CO_2 every 5 minutes for 12 hours. At

each time point and stage position a stack of 3 z-planes was taken, spaced 4µm apart in the brightfield (0.05 light intensity, 125ms exposure time) and Cy5 (0.005 light intensity, 50ms exposure time) channels.

Maximum projections of live cell imaging data were used to quantify the times at which nuclear envelope breakdown (NEBD), metaphase, and anaphase occurred based on DNA morphology. Cells seeded for Western blot were harvested 7 hours after thymidine washout.

RNA sequencing: Total RNA purification and on-column DNAseI-treatment were performed using the RNeasy Mini kit (QIAgen). RNA concentration was assessed using the NanoDrop ND-1000 (Labtech) and RNA quality using the Agilent 2100 Bioanalyzer (Agilent). RNA-sequencing (3 independent experiments), sequence annotation and normalization were performed at the Oxford Genomics Centre (WTCHG, Oxford, UK). Polyadenylated transcript enrichment and strand specific library preparation was completed using NEBNext Ultra II mRNA kit (NEB) following manufacturer's instructions. Libraries were amplified on a Tetrad (Bio-Rad) using in-house unique dual indexing primers (based on DOI: 10.1186/1472-6750-13-104). Individual libraries were normalised using Qubit, and the size profile was analysed on the 2200 or 4200 TapeStation. Individual libraries were normalised and pooled together accordingly. The pooled library was diluted to ~10nM for storage. The 10nM library was denatured and further diluted prior to loading on the sequencer. Paired end sequencing was performed using a HiSeq4000 75bp platform (Illumina, HiSeq 3000/4000 PE Cluster Kit and 150 cycle SBS Kit), generating a raw read count of 20-25 million reads per sample. The sequencing quality was assessed with FASTQC (http://www.bioinformatics.babraham.ac.uk/projects/fastqc/) and the raw sequencing reads were mapped to the human genome hg19 using STAR version 2.5.1.

Single cell RNA-sequencing (scRNA-seq): For scRNA-seq, AT samples were homogenised, collagenase-digested and centrifuged to isolate the stromovascular fraction (SVF). SVF cells were re-suspended in FBS containing 10% DMSO and stored in liquid nitrogen. ScRNA-seq libraries were generated using 10x Genomics Chromium Single Cell 30 Library & Gel Bead and i7 Multiplex kits. Sequencing was performed on an Illumina NextSeq500 using a 75-bp paired-end kit. Sequence was aligned and demultiplexed using Cell Ranger (10x Genomics) and demuxlet [6], and the expression matrix was generated. The R package Seurat (https://satijalab.org/seurat) was used for quality control, data normalization and scaling, clustering and annotation of cell types. The data were obtained from a 10x scRNA-seq dataset containing 54708 subcutaneous abdominal and gluteal stromal vascular cells. Here, we show only the look-up for LRP5 to confirm cellular distribution of gene expression, the rest of the data are unpublished.

Statistical analysis comparing *LRP5* variant carriers and controls: Studies comparing *LRP5* variant carriers and controls were as follows: for every GoF (total n=6) or LoF case (total n=23) the ten most closely age-, sex-, and BMI-matched controls were chosen from the OBB. Clinical characteristics of these participants are shown in **Supplementary Tables 3** and **5**. P-values for both tables were obtained from t-test for normally distributed variables and Mann-Whitney test for skewed variables. To determine the variation in the mean difference within each paired cluster, all clinical outcomes were standardized using the mean and standard deviation (SD) within each cluster [Z-outcome = (individual value - mean of the outcome within the cluster)/SD of the cluster], so that within each cluster the mean is approximated to 1 and SD is zero and all variables assume normal distribution. The difference in mean and 95% confidence interval within each cluster was obtained by 2-sample t-test. Results are interpreted as number of SDs the mean difference in outcome of the case varies from the controls. To study the effects of ageing in *LRP5* GoF vs. age- and BMI-matched control females, for each *LRP5* GoF female, we selected ten BMI-matched control females who were 5-20 years younger. We then determined the difference in mean and

95% confidence interval within each cluster for *LRP5* GoF, age-matched controls and younger BMI-matched females as above.

Microscopy and Western blot densitometry

Micrographs were captured on a Nikon Eclispe TS100 inverted microscope with the GXCapture-T software. Fluorescence images of AdipoRed-stained cells were taken through a DM500 BA515 B-2A (EX450-490) filter. All images from a single experiment were taken at the same magnification with a fixed exposure time.

Protein quantification was performed using the Bio-Rad *DC* Protein assay (Bio-Rad) and a VERSAmax microplate reader (Molecular Devices). Western blot protein densitometry was performed using the ImageJ (v1.53e) software.

SUPPLEMENTARY REFERENCES:

1 Karpe, F. *et al.* Cohort Profile: The Oxford Biobank. *Int J Epidemiol* **47**, 21-21g, doi:10.1093/ije/dyx132 (2018).

2 Gregson, C. L. *et al.* Analysis of body composition in individuals with high bone mass reveals a marked increase in fat mass in women but not men. *J Clin Endocrinol Metab* **98**, 818-828, doi:10.1210/jc.2012-3342 (2013).

3 Dang, L. T. *et al.* Receptor subtype discrimination using extensive shape complementary designed interfaces. *Nat Struct Mol Biol* **26**, 407-414, doi:10.1038/s41594-019-0224-z (2019).

4 Miao, Y. *et al.* Next-Generation Surrogate Wnts Support Organoid Growth and Deconvolute Frizzled Pleiotropy In Vivo. *Cell Stem Cell* **27**, 840-851 e846, doi:10.1016/j.stem.2020.07.020 (2020).

5 Thermofisher. <https://www.thermofisher.com/document-connect/document-connect.html?url=https://assets.thermofisher.com/TFS-Assets/BID/Application-Notes/potent-activation-wnt-pathway-fusion-protein-app-note.pdf> (

6 Kang, H. M. *et al.* Multiplexed droplet single-cell RNA-sequencing using natural genetic variation. *Nat Biotechnol* **36**, 89-94, doi:10.1038/nbt.4042 (2018).
