# Supplementary material for: LRP5 promotes adipose progenitor cell fitness and adipocyte insulin sensitivity
Source: Commun Med (Lond). 2025 Feb 25;5:51. doi: 10.1038/s43856-025-00774-1 (PMC11862225; doi:10.1038/s43856-025-00774-1)
Supplement: Supplementary file 3 — Description of Additional Supplementary Files [file 43856_2025_774_MOESM3_ESM.pdf]

Description of Additional Supplementary Files

File name: Supplementary Data 1

File description: Biochemical and body compostion profile of individuals with LRP5-loss of function variants within cluster of age, sex and BMI-matched controls

File name: Supplementary Data 2

File description: Comparison of tissue-specific LRP5 and VCP gene expression in different age groups using GTEx data.

File name: Supplementary Data 3

File description: Source data for Figures 1-7 and gene counts for RNA sequencing data.
