# Supplementary material for: LRP5 promotes adipose progenitor cell fitness and adipocyte insulin sensitivity
Source: Commun Med (Lond). 2025 Feb 25;5:51. doi: 10.1038/s43856-025-00774-1 (PMC11862225; doi:10.1038/s43856-025-00774-1)
Supplement: Supplementary file 7 — Reporting summary [file 43856_2025_774_MOESM7_ESM.pdf]

nature portfolio

Corresponding author(s): Constantinos Christodoulides

Last updated by author(s): 12 February 2025

Reporting Summary

Nature Portfolio wishes to improve the reproducibility of the work that we publish. This form provides structure for consistency and transparency in reporting. For further information on Nature Portfolio policies, see our Editorial Policies and the Editorial Policy Checklist.

Statistics

For all statistical analyses, confirm that the following items are present in the figure legend, table legend, main text, or Methods section.

n/a	Confirmed	
☐	☒	The exact sample size (*n*) for each experimental group/condition, given as a discrete number and unit of measurement
☐	☒	A statement on whether measurements were taken from distinct samples or whether the same sample was measured repeatedly
☐	☒	The statistical test(s) used AND whether they are one- or two-sided<br>*Only common tests should be described solely by name; describe more complex techniques in the Methods section.*
☐	☒	A description of all covariates tested
☐	☒	A description of any assumptions or corrections, such as tests of normality and adjustment for multiple comparisons
☐	☒	A full description of the statistical parameters including central tendency (e.g. means) or other basic estimates (e.g. regression coefficient) AND variation (e.g. standard deviation) or associated estimates of uncertainty (e.g. confidence intervals)
☒	☐	For null hypothesis testing, the test statistic (e.g. *F*, *t*, *r*) with confidence intervals, effect sizes, degrees of freedom and *P* value noted<br>*Give P values as exact values whenever suitable.*
☒	☐	For Bayesian analysis, information on the choice of priors and Markov chain Monte Carlo settings
☒	☐	For hierarchical and complex designs, identification of the appropriate level for tests and full reporting of outcomes
☐	☒	Estimates of effect sizes (e.g. Cohen's *d*, Pearson's *r*), indicating how they were calculated

Our web collection on statistics for biologists contains articles on many of the points above.

Software and code

Policy information about availability of computer code

Data collection	Veritas Microplate Luminometer (Turner Biosystems), Pherastar microplate reader (BMG Labtech), VERSAmax microplate reader (Molecular Devices, QuantStudio 7 Flex (QuantStudio Real-Time PCR software v1.3, Applied Biosystems), GXCapture-T software on a GXCAM-U3PRO-6.3 digital camera & a Nikon Eclypse TS100 inverted microscope, Beckman LS6500 Multipurpose Scintillation Counter (Beckman), ImageJ, Konica SRX-101A X-ray developer, enCORE v14.1
Data analysis	GraphPad Prism v10.1.3, IBM SPSS v29.0.0, DESeq2 v1.34.0, TwoSampleMR v0.5.7, R v4.3.1, Metascape, iRegulon in Cytoscape

For manuscripts utilizing custom algorithms or software that are central to the research but not yet described in published literature, software must be made available to editors and reviewers. We strongly encourage code deposition in a community repository (e.g. GitHub). See the Nature Portfolio guidelines for submitting code & software for further information.

Data

Policy information about availability of data

All manuscripts must include a data availability statement. This statement should provide the following information, where applicable:

- Accession codes, unique identifiers, or web links for publicly available datasets
- A description of any restrictions on data availability
- For clinical datasets or third party data, please ensure that the statement adheres to our policy

Source data for Figures 1-7 and gene counts for RNA sequencing data are available in Supplementary Data 3. Other data and resource generated during the current

study are available from the corresponding author upon reasonable request. All MR analyses were conducted using publicly available data with links to GWAS sources available in Supplementary Table 2.

Human research participants

Policy information about studies involving human research participants and Sex and Gender in Research.

Reporting on sex and gender	The findings in this study applies to both males and females, unless otherwise stated. Sex of participants were determined by self-reporting and confirmed by genotyping. Gender data was not collected. Where analyses involves data from both sexes, analyses have been adjusted for sex. Overall numbers are provided below.
Population characteristics	The Oxford BioBank (OBB) comprises a randomised, age-stratified sample obtained from Oxfordshire and the Thames Valley in the U.K.. The Thames Valley Primary Care Agency has enabled random recruitment by providing lists of Oxfordshire residents registered with a local general practitioner and aged 30–50 years. Individuals with a previous diagnosis of myocardial infarction or heart failure currently on treatment; untreated malignancy; other ongoing systemic diseases, and pregnant women were excluded from participation. The OBB recruitment began in 1999 and included 7640 individuals (4316 women and 3324 men) as of October 2016. OBB subjects were genotyped on the Illumina Human Exome BeadChip and Affymetrix UK Biobank Axiom arrays. Genotype imputation was performed using the Affymetrix UK Biobank Axiom array with Haplotype Reference Consortium (HRC), 1000Genome and UK10K reference panels using IMPUTE2 software.<br><br>The HBM study is a UK-based multi-centered observational study of adults with unexplained HBM identified by screening 335,115 DXA scans from 13 UK DXA databases from which 258 HBM probands with BMD Z-score ≥+3.2 were identified. All participants were clinically assessed by one doctor using a standardized structured history and examination questionnaire, after which total-body Lunar prodigy DXA scans were performed (PMID: 23337721). Written informed consent was collected for all, in line with the Declaration of Helsinki. Participants were excluded if they were aged <18 years, pregnant, or unable to provide written informed consent for any reason. This study was approved by the Bath Multicenter Research Ethics Committee (REC) and at each NHS Local REC. Cases with HBM LRP5 mutations were identified by targeted sequencing of exons 2-4 of LRP5 (i.e. the sites of previously described HBM cases).
Recruitment	An invitation letter along with the study information and response sheet were sent to all participants. Individuals who expressed willingness to enroll in this study were contacted by telephone or email, in order to convey a brief overview of the study aims and objectives, by trained research nurses.
Ethics oversight	All studies were approved by the Oxfordshire Clinical Research Ethics Committee and all volunteers gave written, informed consent (IRAS Ethics approval 18/SC/0588).

Note that full information on the approval of the study protocol must also be provided in the manuscript.

Field-specific reporting

Please select the one below that is the best fit for your research. If you are not sure, read the appropriate sections before making your selection.

☒ Life sciences ☐ Behavioural & social sciences ☐ Ecological, evolutionary & environmental sciences

For a reference copy of the document with all sections, see nature.com/documents/nr-reporting-summary-flat.pdf

Life sciences study design

All studies must disclose on these points even when the disclosure is negative.

Sample size	No formal sample size calculations were performed. Instead sample sizes were determined based on our own previous experience and publications of similar studies.
Data exclusions	No data was excluded, except for one extreme outlier for experiments in Fig. 5a and Fig.5b that was confirmed in by an outlier test, and qRT-PCR from one sample in Fig 7b due to RNA degradation.
Replication	All in vitro studies were replicated in at least 3 independent experiments, unless otherwise stated. In the case where an experiment was only replicated twice, the results were conclusive.
Randomization	This is not relevant as it is not a clinical study.
Blinding	There was no blinding.

Reporting for specific materials, systems and methods

We require information from authors about some types of materials, experimental systems and methods used in many studies. Here, indicate whether each material, system or method listed is relevant to your study. If you are not sure if a list item applies to your research, read the appropriate section before selecting a response.

Materials & experimental systems

n/a	Involved in the study
☐	☒ Antibodies
☐	☒ Eukaryotic cell lines
☒	☐ Palaeontology and archaeology
☒	☐ Animals and other organisms
☒	☐ Clinical data
☒	☐ Dual use research of concern

Methods

n/a	Involved in the study
☒	☐ ChIP-seq
☒	☐ Flow cytometry
☒	☐ MRI-based neuroimaging

Antibodies

Antibodies used	From Santa Cruz Biotechnology: Actin (I-19)-HRP goat pAb (sc-1616), cyclin D1 (M-20) rabbit pAb (sc-718): from Cell Signaling Technology: LRP5 (D80F2) rabbit mAb (#5731), pAKT-S473 rabbit pAb (#9271), total AKT rabbit pAb (#9272), cMYC (E5Q6W) rabbit mAb (#18583); from Millipore: Active beta-catenin (clone 8E7) mouse mAb (#05-665); from Abcam: alpha-tubulin rabbit pAb (ab15246); from Enzo: Mono- and polyubiquitinylated conjugates recombinant monoclonal antibody (UBCJ2) (HRP conjugate) (ENZ-ABS840HRP-0100); from BioLegend Inc: VCP mouse mAb (#870902)
Validation	All primary antibodies used have been validated by manufacturers and by citations in publications.

Eukaryotic cell lines

Policy information about cell lines and Sex and Gender in Research

Cell line source(s)	DFAT cell lines were generated in-house from immortalised (human telomerase reverse transcriptase and HPV-E7 oncoprotein) adipose progenitors derived from abdominal and gluteal fat biopsies of a male subject. HEK293 cells were purchased from ATCC.
Authentication	We have used gene expression signatures (by Taqman PCR, RNA-seq) and functional studies (PMID: 284525920) to authenticate the depot origin of the cell lines.
Mycoplasma contamination	All cell-lines have been tested routinely and confirmed to be free of mycoplasma contamination.
Commonly misidentified lines (See ICLAC register)	None.
